# Supplementary material for: Macrodiolide Formation by the Thioesterase of a Modular Polyketide Synthase
Source: Angew Chem Weinheim Bergstr Ger. 2015 Mar 6;127(17):5321–4. doi: 10.1002/ange.201500401 (PMC4535664; doi:10.1002/ange.201500401)
Supplement: Supplementary file 1 — miscellaneous_information [file ange0127-5321-sd1.pdf]

Angewandte Chemie
Eine Zeitschrift der Gesellschaft Deutscher Chemiker

Supporting Information

Macrodiolide Formation by the Thioesterase of a Modular Polyketide Synthase**

*Yongjun Zhou, Patrícia Prediger, Luiz Carlos Dias, Annabel C. Murphy, and Peter F. Leadlay**

ange_201500401_sm_miscellaneous_information.pdf

Supporting Information

Table of Contents

1. Supplementary Methods

1.1. High pressure liquid chromatography (HPLC) and mass spectrum (MS) procedures...........................3

1.2. Bacterial strains and culture conditions...........................3

1.3. DNA manipulation...........................3

1.4. Expression and purification of recombinant proteins...........................4

1.5. Enzyme assays...........................4

1.6. Purification of the products generated from Ela-TE assays...........................4

1.7. Analytical ultracentrifugation (AUC) analysis...........................5

2. Tables and Figures

Table S1. Plasmid construction and oligonucleotide primers...........................5

Table S2. The protein molecular weights measured by HPLC-MS...........................5

Figure S1. HPLC–MS analysis and SDS-PAGE gel of the purified proteins...........................6

Figure S2. AUC analysis of Ela-TE...........................6

Figure S3. The production time-plot of compound **4** and **5** generated by Ela-TE...........................7

Figure S4. The linear dimer intermediates generated by Ela-TE from the mixture substrates...........................7

Figure S5. Multiple alignment of the functional TE domains expressed in *E. coli*...........................8

Figure S6. The modeled structure of Ela-TE...........................8

3. Compound Synthesis Procedures and the NMR and HRMS Confirmation

3.1. Tetraketide **2b** (Scheme S1)...........................10

3.2. Pentaketide **3b** (Scheme S2)...........................16

4. NMR and HRMS Elucidation of the Compounds Generated by Ela-TE

4.1. Compound **4**...........................23

4.2. Compound **5**...........................24

4.3. Compound **6**...........................24

5. Selected NMR Spectra

5.1. Compound **4**...........................26

5.2. Compound **5**...........................28

5.3. Compound **6**...........................31

5.4. Compound **3b**...........................34

5.5. Compound **2b**...........................36

6. Supplementary References...........................37

1. Supplementary Methods

1.1. High pressure liquid chromatography (HPLC) and mass spectrum (MS) procedures.

HPLC-MS analysis was performed using an HPLC (Agilent Technologies 1200) coupled to a Thermo Fisher LTQ mass spectrometer fitted with an electrospray ionization (ESI) source. The HPLC was fitted with a Prodigy 5µ C18 column (4.6 × 250 mm, Phenomenex) column. A solvent system of MeCN (acetonitrile) and water both containing 0.1% formic acid (v/v) was used. Samples were eluted at a flow rate 0.7 mL min^{-1} with a linear gradient of 30 to 95 % of MeCN over 30 min for Ela-TE assay analysis. The mass spectrometer was run in positive ionization mode, scanning from m/z 200 to 2000 and normalized collision energy of 35%.

Mass analysis of recombinant proteins was carried out by HPLC-MS using a Jupiter 5µ C4 column (300Å, 250 x 2 mm, Phenomenex). A solvent system of MeCN and water both containing 0.1% trifluoroacetic acid was used at a flow rate of 0.3 ml min^{-1} with a linear gradient of 5 to 35% of MeCN over 5 min, then 35 to 95% of MeCN over 15 min. The LTQ mass spectrometer was operated in positive ion mode, scanning from m/z 600 to 2000.

Preparative HPLC purification was performed on Agilent Technologies 1200, using C18 column (100Å, 250 × 21.20 mm, 10 micron, Phenomenex) at a flow rate of 15 mL min^{-1}. Sample injection volume was 200 or 500 µL.

ESI high resolution MS (ESI-HR-MS) was carried out on Thermo Fisher Orbitrap with 30,000 resolution.

HPLC-MS data were processed and deconvoluted using Xcalibur (v. 1.1) (Thermo Finnigan).

1.2. Bacterial strains and culture conditions.

Streptomyces violaceusniger DSM 4137, the producer strain of elaiophylin [1], was grown in TSBY medium (3% tryptone soy broth, 10.3% sucrose, 0.5% yeast extract) at 30 °C, 200 rpm, 250 ml flask with 30 ml medium and metal spring. *E. coli* strains were grown in Luria-Bertani (LB) broth (1% tryptone, 0.5% yeast extract, 0.5% NaCl) or LB agar medium (2% agar) at 37°C with 50 µg mL^{-1} kanamycin.

1.3. DNA manipulation.

Bacterial strains, plasmids and oligonucleotides (Invitrogen) used in this work are summarised in Tables S1 and S2 respectively. Restriction endonucleases and T4 DNA ligase were purchased from New England Biolabs. Chemicals were purchased from Sigma-Aldrich.

Plasmid DNA was isolated from an overnight culture using the Plasmid Mini Kit I (Omega BioTek) according to the manufacturer's protocol. PCR amplifications were carried out using a Mastercycler (Eppendorf) and the *Phusion* High-Fidelity PCR Master Mix from New England Biolabs (for cloning), or BioMix Red from Bioline (for screening purposes). Genomic DNA isolated from 3 d mycelium was used as template. DNA sequencing was carried out by the DNA Sequencing Facility in the Department of Biochemistry, University of Cambridge.

1.4. Expression and purification of recombinant proteins.

The expression plasmids pYJ4 and pKJW63 [3] (Table S1) were each introduced into BL21(DE3) and BL21 (DE3) plysS. A single colony was inoculated into 5 mL of LB medium containing 50 μg mL^{-1} kanamycin and grown overnight at 37 °C, 250 rpm. 2 ml of the culture was inoculated into 1 L LB medium containing 50 μg mL^{-1} kanamycin and incubated at 37 °C, 200 rpm until A600 reached 0.6~0.8 before adding 200 μL of 1 M isopropyl-*β-D*-thiogalactopyranoside and incubating at 22 °C for 15 h to induce protein expression. Cells were harvested by centrifugation at 8,000 rpm for 6 min, and then resuspended in lysis buffer (50 mM *tris*-HCl, 0.3 M NaCl, pH 7.2) and lysed by sonication. The total lysate was centrifuged at 17,000 rpm for 25 min, and the supernatant was filtrated with 0.45 μm filter before loading on a His-Bind column (1 mL bed volume), which was pre-charged with nickel ions and equilibrated with lysis buffer. The column was washed with 10 column volumes of lysis buffer. Bound proteins were eluted with a step gradient of increasing imidazole concentration (10, 20, 40, 80, 100, 150, 200, 250 and 500 mM in binding buffer). Ela-TE, and DEBS-TE were eluted respectively at imidazole concentration of 20 and 80 mM. The protein solutions were concentrated, and the buffer was exchanged into 100 mM potassium phosphate buffer pH 8.2, using Amicon Ultra-4 concentrators (Millipore) fitted with a filter of 10 KD pore size. The protein yield of Ela-TE is 6 mg L^{-1} and the yield of DEBS-TE is 0.5 mg L^{-1}. The purified proteins were examined by 4 - 12% Bis-Tris Gel (Novex) analysis and HPLC-MS (Figure S1 and Table S2). Protein concentration was measured by NanoDrop 1000 Spectrophotometer and determined by the mean value of three 2× dilutions samples (adjust to 1 to 6 mg mL^{-1}).

1.5. Enzyme assays.

The standard assays were performed with 50 μl system of 3 mM substrate, 10% (v/v) dimethyl sulfoxide, 0.1 M potassium phosphate buffer (pH8.2), 40 μM enzyme, 5 h incubation at 20 °C.

To accumulate the product **4** and **5**, Ela-TE assay was scaled up to 10 ml with 50 μM enzyme, 8 mM **3b** substrate and incubated at 20 °C for 12 h.

To generate product **6**, both **2b** and **3b** substrates were used by 3 mM concentration, and the assay was scaled up to 23 ml with 40 μM enzyme and incubated at 20 °C for 36 h.

1.6. Purification of the products generated from Ela-TE assay.

Enzyme was removed efficiently from the assay solution by adding 4 volume of MeCN and subsequently 10 min centrifuge. The supernatant was evaporated to remove MeCN and the remaining aqueous phase was extracted with 0.5 volume of ethyl acetate three times. The organic extract was dried and redissolved into 2 ml methanol as the sample injected to preparative-HPLC.

To purify the products **4**, **5** and **6** generated from Ela-TE assay by using preparative-HPLC, water and MeCN were used as elution solvents with gradient of 30 to 95% MeCN over 30 min. UV detection was set as Sig=260 Ref=360. The retention times for **4**, **5** and **6** are respectively 19.8, 28.5 and 29.9 min. The target fragments were combined and evaporated to remove MeCN. Then the remaining aqueous solution was extracted with 0.5 volume of ethyl acetate three times. Finally, 1.5 mg **5** (white powder), ~ 0.5 mg **4** (faint

yellow oil) and ~ 0.8 mg **6** (white powder) were respectively obtained after drying the organic extract. All the products were confirmed by ESI-HR-MS and ^{1}H, ^{13}C and 2D NMR **(Supporting Information 4)**.

1.7. Analytical ultracentrifugation (AUC) analysis.

AUC-Sedimentation velocity experiments were conducted with an Optima XL-I (Beckman Coulter) centrifuge using an An60 Ti four-hole rotor. Standard double-sector Epon centrepieces equipped with sapphire windows contained 400 μL of Ela-TE at 3 mg/ml. Interference data were acquired in the continuous mode at time intervals of 170 s and rotor speed of 45,000 rpm, at a temperature of 20 °C with systematic noise subtracted, without averaging. The density and viscosity of the buffer (0.1 M potassium phosphate, pH 8.2) and the partial specific volume of the protein were calculated using Sednterp [7]. Multi-component sedimentation coefficient distributions were obtained from 128 scans by direct boundary modelling of the Lamm equation using Sedfit v.14.1 [8].

2. Tables and Figures

Table S1. Plasmid construction and oligonucleotide primers.

Plasmid	Protein	Constructions
**pYJ4**	Ela-TE	The DNA fragment encoding Ela-TE was amplified from genomic DNA of *S. violaceusniger* DSM 4137[1] by the primer Ela-TE-S: 5' TTTAACTTTAAGAAGGAGATATACATGTCCGACCTGGCCGCGGCCAAGCA 3', Ela-TE-A: 5' ATCTCAGTGGTGGTGGTGGTGGTGCTCGGCGCCGTCGCGCTCCAG 3'. The PCR product was introduced into the NcoI and XhoI sites of pET28b(+) by isothermal assembly[9]; The C-His-tag was introduced by the primer.
**pKJW63[3]**	DEBS-TE	The C-terminal thioesterase/cyclase DEBS-TE derived from the erythromycin PKS was expressed based on pET29b(+).

Table S2. The protein molecular weights measured by HPLC-MS.

Protein	Protein expression host	Molecular weight		
		Calc.	Det.	Error
**Ela-TE**	BL21(DE3)	33715	33710	-5
**DEBS-TE ***	BL21 (DE3) plysS	31047	31043	-4

* The protein is expressed with N-terminal methionine.

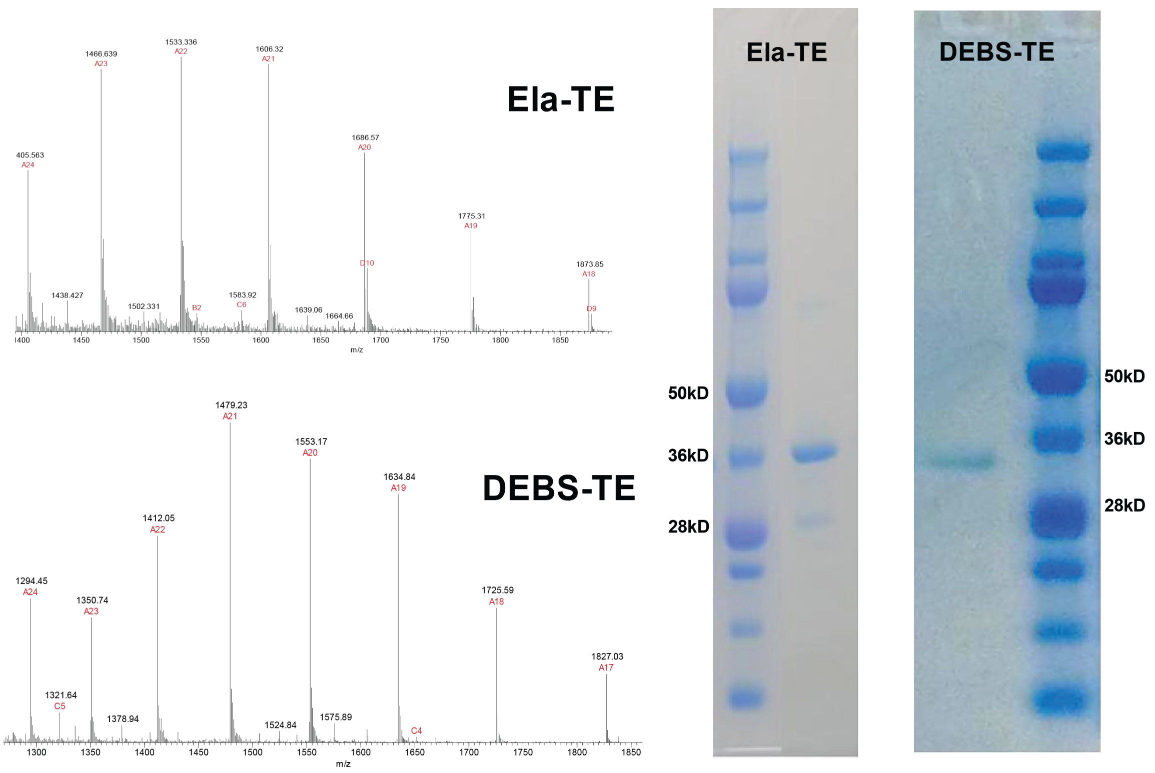

Figure S1. HPLC–MS analysis (left part) and SDS-PAGE gel (right part) of the purified proteins.

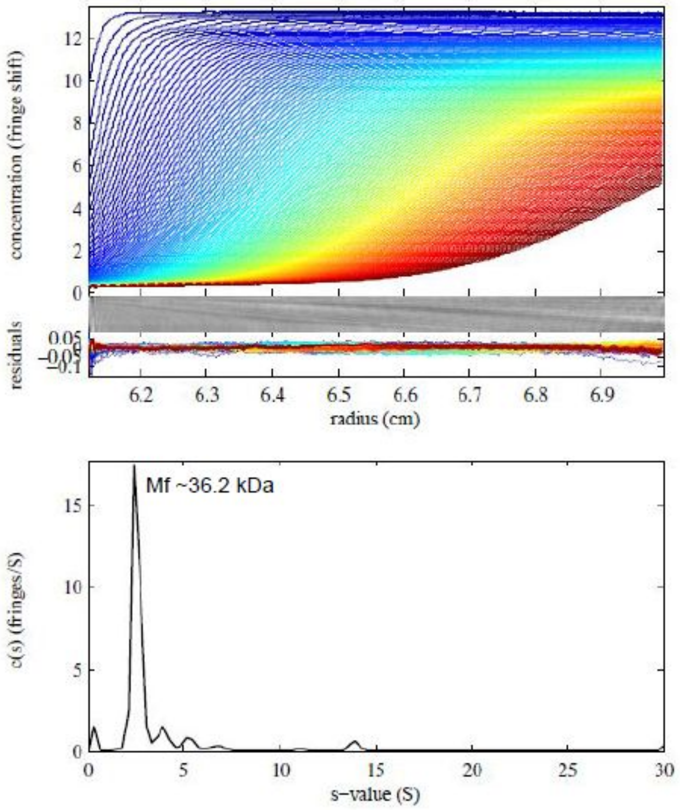

Figure S2. AUC analysis of Ela-TE. The residuals are from the fit with the continuous c(s) distribution model. Component sedimentation coefficient distribution shows Ela-TE is predominately monomeric with an

so20,w value of 2.73 S, assuming a uniform frictional ratio of Fk,w = 1.400263, although low populations of other oligomer species are evident. The final r.m.s.d. was 0.011475. (see method, Supporting Information 1.7)

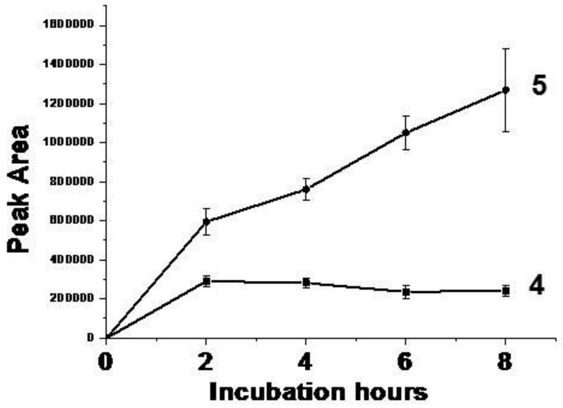

Figure S3. The production time-plot of compound **4** and **5** generated by Ela-TE from substrate **2b**.
The time plot was made based on x-axis hours and y-axis peak area of extracted mass from total ion of chromatography of HPLC-MS analysis. Three parallel assays were analyzed to make each time point of the curves. Clear background was confirmed by non-enzyme control.

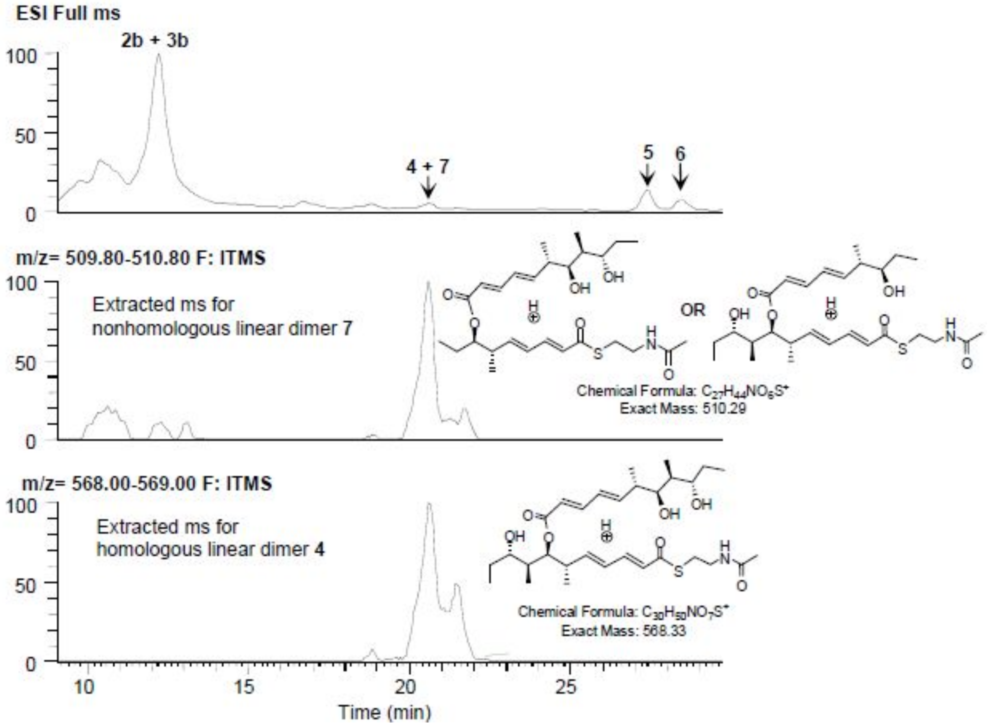

Figure S4. The linear dimer intermediates generated by Ela-TE from the mixture substrates.
When an equimolar mixture of **2b** and **3b** was incubated with Ela-TE, two linear dimer intermediates, homologous **4** and nonhomologous **7**, were detected by HPLC-MS at 20.5 min retention time.

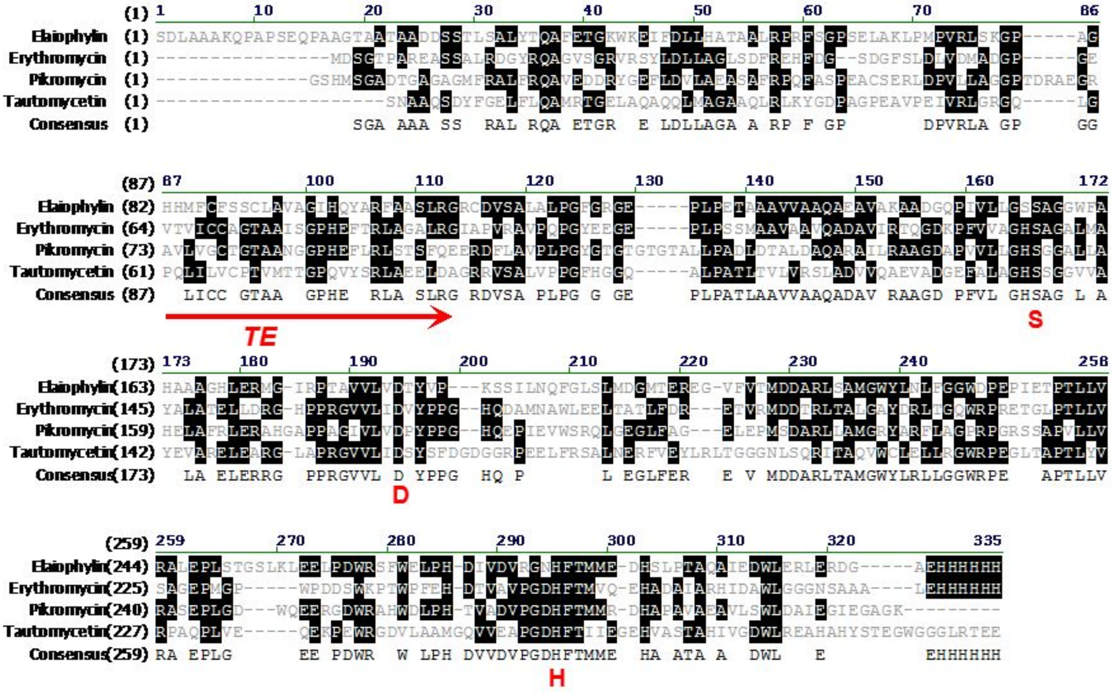

Figure S5. Multiple alignment of the functional TE domains expressed in *E. coli*.
Ela-TE (elaiophylin) was investigated in this work. The other three TEs respectively derived from erythromycin [6], pikromycin [4], and tautomycetin [5] were characterized by crystal structure. The start region of the TE domains and the active residues are all labeled.

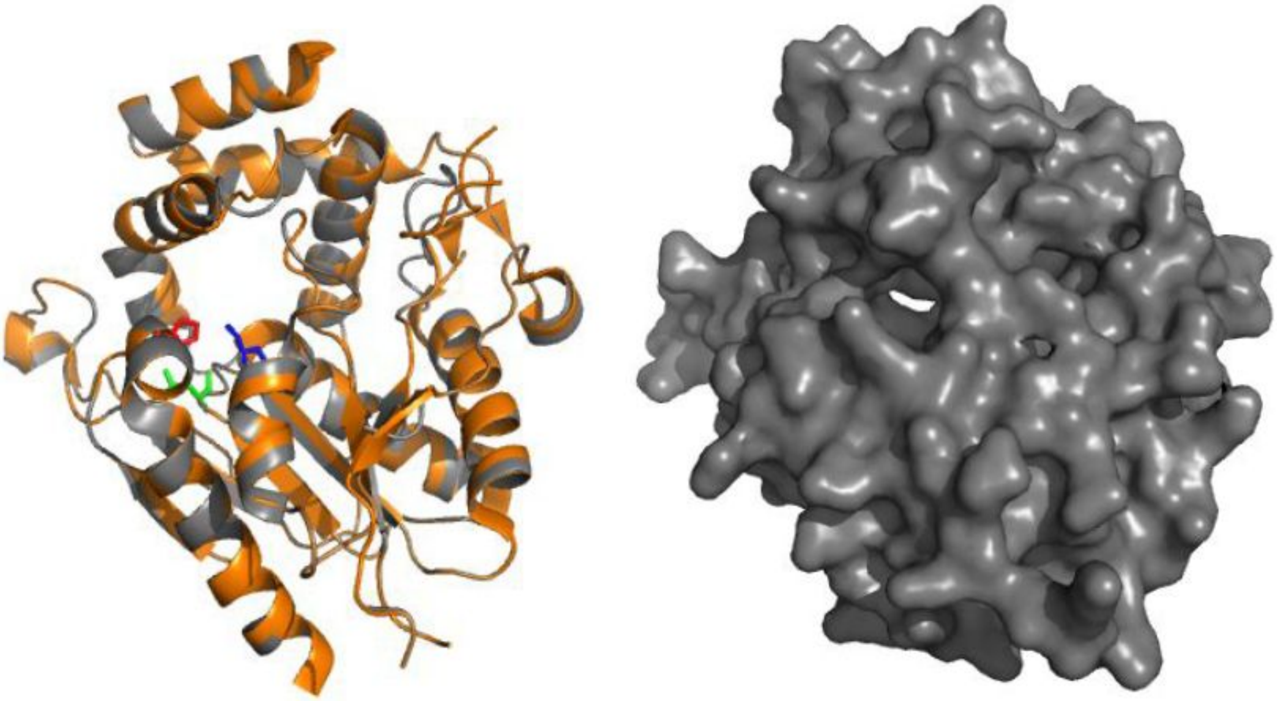

Figure S6. The modeled structure of Ela-TE (the figure was made by PyMOL).
The structures of Ela-TE is modeled based on the template of pikromycin thioesterase (c2h7xA). a) the superimposing model of pikromycin TE (orange) and Ela-TE (grey). the triplet catalytic sites of Ela-TE are indicated, blue, Ser; green, Asp; red, His. b) the model of Ela-TE surface with view of the substrate channel.

3. Compound Synthesis Procedures and the NMR and HRMS Confirmation.

General Remarks: All reactions, unless otherwise specified, were performed under an atmosphere of argon. Dichloromethane (DCM), toluene, triethylamine (Et_3N), diisopropylethylamine (DIPEA), acetonitrile (CH_3CN), methanol (MeOH), and dimethyl sulfoxide (DMSO) were treated with calcium hydride and distilled prior to use. Tetrahydrofuran (THF) and ethyl ether (Et_2O) were treated with calcium hydride, distilled, and treated with sodium and benzophenone prior to use. Acetic acid (AcOH) was distilled in the presence of acetic anhydride and $KMnO_4$. Oxalyl chloride was distilled immediately prior to use. Camphorsulfonic acid (CSA) was recrystallized from ethyl acetate and then dried under *vacuum*. Other reagents were used without pretreatment. Compounds were purified by flash column chromatography using silica gel (230– 400 mesh) as the stationary phase. The mobile phase used is specified for each experimental procedure. Thin-layer chromatography (TLC) on silica gel 60 and GF (5–40 μm thickness) plates was used for monitoring the progress of reactions. TLC plates were visualized using UV light and then stained with phosphomolybdic acid and heat. The maximum absorbance wavelengths (max) of infrared spectra are presented in wavenumbers (cm^{-1}). The angles of deviation of the polarized light (α) are described as follows: (*c* [g/100 mL], solvent). High-resolution mass spectrometry (HRMS) was performed using electrospray ionization (ESI). ^1H and proton-decoupled ^{13}C NMR spectra were recorded in $CDCl_3$ or [D_4]-methanol at 250 MHz (^1H) and 62.5 MHz (^{13}C); 400 MHz (^1H) and 100 MHz (^{13}C); 500 MHz (^1H) and 125 MHz (^{13}C) or 600 MHz (^1H) and 150 MHz (^{13}C). The chemical shifts (δ) are reported in ppm using the solvent peak as an internal standard ([D_4] methanol at δ = 3.30 ppm and $CDCl_3$ at δ = 7.26 ppm for ^1H NMR spectra, and [D_4]methanol at δ = 49.0 ppm and $CDCl_3$ at δ = 77.0 ppm for ^{13}C NMR spectra). Data are reported as follows: multiplicity (s = singlet, br. s = broad singlet, d = doublet, t = triplet, q = quartet, quint = quintuplet, sext = sextet, dd = doublet of doublets, dt = doublet of triplets, dq = doublet of quartets, ddd = doublet of doublet of doublets, td = triplet of doublets, tt =triplet of triplets, qd = quartet of doublets, or m = multiplet), coupling constant(s) in Hz, integration.

3.1. Tetraketide 2b.

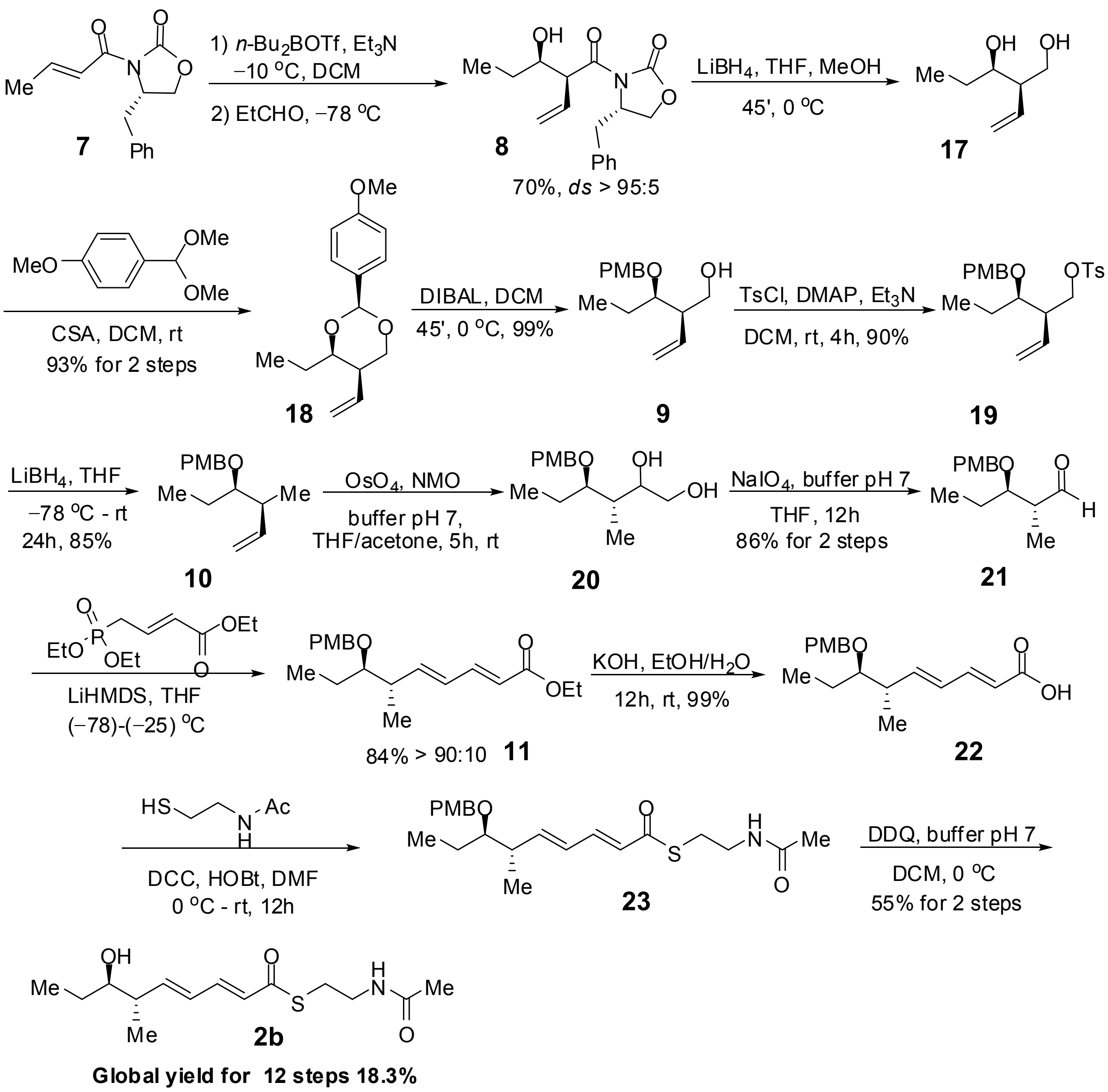

Scheme S1. Substrate **2b** synthesis.

(*S*)-4-benzyl-3-((2*S*,3*R*)-3-hydroxy-2-vinylpentanoyl)oxazolidin-2-one (8)[10]

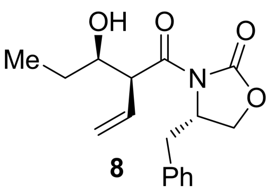

8

A solution of (*S*,*E*)-4-benzyl-3-(but-2-enoyl)oxazolidin-2-one (**7**) (8.16 mmol, 1.99 g) in 43 mL of CH_2Cl_2 was cooled to −78 °C. A solution of dibutylboron triflate (8.91 mmol, 2.01 mL, in 7 mL of CH_2Cl_2) was added to the reaction dropwise via cannula. The mixture was stirred for 5 min and then triethylamine (12.4 mmol, 1.73 mL in 7 mL of CH_2Cl_2) was added slowly. A yellow color quickly developed as the reaction mixture was maintained at −78 °C for 1 h, then warmed to 0 °C, and maintained at that temperature for 20 min. The solution was recooled to −78 ºC and propanal (42.5 mmol, 3.05 mL in 25 mL de CH_2Cl_2) was added dropwise. The solution was stirred at −78 °C for 90 min and then warmed to 0 °C and maintained at that temperature for 1 h. The reaction was quenched by the addition of 8.5 mL of phosphate buffer pH 7

and methanol (24.6 mL). After, a solution of methanol/H_2O_2 30% (2/1, 24.6 mL) was added. The reaction was stirred for 1 h and concentrated *in vacuum*. The organic layer was dissolved in diethyl ether and saved and the aqueous layer was extracted (3 x with 40 mL of diethyl ether). The combined organic layers were washed twice with brine, dried over $MgSO_4$, filtered, and concentrated *in vacuum*. The crude reaction product was purified by flash column chromatography (silica gel, EtOAc/hexanes 15/85) to give 1.68 g of compound **8** (70%, dr = > 95:5) as a pale yellow oil. R*f* = 0.46 (30% EtOAc in hexane). $[\alpha]^{20}_D$ –3.0 (c 2.9; $CHCl_3$). ^1H NMR (400 MHz, $CDCl_3$): 7.26–7.18 (m, 3H), 7.12 (d, J = 8.0 Hz, 2H), 5.95 (dd, J_1 = 17.0 Hz, J_2 = 10.5 Hz, 1H), 5.34–5.31 (m, 2H), 4.67–4.62 (m, 1H), 4.51 (dd, J_1 = 9.0 Hz, J_2 = 3.7 Hz, 1H), 4.16–4.08 (m, 2H), 3.84–3.83 (m, 1H), 3.16 (dd, J_1 = 13.4 Hz, J_2 = 3.0 Hz, 1H), 2.96 (d, J = 2.1 Hz, 1H), 2.69 (dd, J_1 = 13.4 Hz, J_2 = 9.0 Hz, 1H), 1.51–1.41 (m, 2H), 0.90 (t, J = 7.5 Hz, 3H) ppm. ^{13}C NMR (100 MHz, $CDCl_3$): 174.2; 152.8; 134.8; 131.0; 129.3; 128.8; 127.3; 121.2; 73.0; 65.9; 55.0; 51.7; 37.4; 27.0; 10.0 ppm. IR υ_{max} (film): 3521, 3020, 2969, 2933, 2879, 1777, 1689, 1386, 1354, 1214, 747, 668 cm^{-1}.
HRMS calc. for $C_{17}H_{21}NO_4Na^+$: 326.1363. Found: 326.1354. Error (ppm): –2.82.

(2*S*,4*R*,5*R*)-4-ethyl-2-(4-methoxyphenyl)-5-vinyl-1,3-dioxane (18)

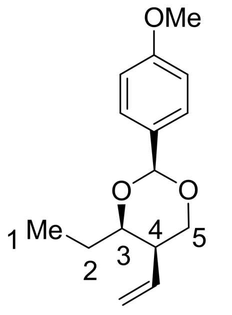

18

A solution of aldol adduct **8** (9.24 mmol, 2.8 g) in THF (37 mL) and MeOH (0.4 mL) was cooled to 0 ºC. Then, $LiBH_4$ (10.2 mmol, 5.1 mL of the 2.0 M solution in THF) was added dropwise. The reaction was stirred for 45 min at this temperature. The reaction was quenched by the addition of saturated aqueous solution of sodium and potassium tartrate (40 mL). After, the reaction media was stirred for 15 min at room temperature. The solvents were removed *in vacuum* and the aqueous layer was extracted with CH_2Cl_2 (3 x 30 mL). The combined organic layers were washed twice with brine, dried over $MgSO_4$, filtered, and concentrated *in vacuum*. The product **17** was isolated as a white solid and was used in the following step without purification. To a solution of the obtained white solid in CH_2Cl_2 (22.5 mL) was added dropwise at room temperature a solution of 4-methoxybenzaldehyde diethyl acetal (10.16 mmol, 2.13 g) in CH_2Cl_2. Catalytic amounts of CSA were added. The reaction was stirred for 3 h and was quenched with saturated aqueous solution of $NaHCO_3$ (100 mL) and diluted with CH_2Cl_2 (60 mL). The organic layer was saved and the aqueous layer was extracted with CH_2Cl_2. The combined organic layers were washed with brine (2 x 50 mL) and dried over $MgSO_4$, filtered and concentrated *in vacuum*. The crude reaction product was purified by flash column chromatography (silica gel, EtOAc/hexanes 5/95) to give 2.13 g of compound **18** (93% for 2 steps) as colorless oil. R*f* = 0.60 (20% EtOAc in hexane). $[\alpha]^{20}_D$ –7.0 (c 2.2, $CHCl_3$). ^1H NMR (500 MHz, $CDCl_3$): 7.44 (d, J = 9.1 Hz, 2H), 6.89 (d, J = 9.1 Hz, 2H), 6.33 (dd, J_1 = 17.0 Hz, J_2 = 10.0 Hz, 1H), 5.51 (s, 1H), 5.21 (dd, J_1 = 10.0 Hz, J_2 = 2.0 Hz, 1H), 5.16 (dd, J_1 = 17.0 Hz, J_2 = 2.0 Hz, 1H), 4.13–4.12 (m, 2H), 3.82–3.76 (m, 4H), 2.09 (dq, J_1 = 10.0 Hz, J_2 = 2.0 Hz, 1H), 1.66–1.58 (m, 1H), 1.50–1.41 (m, 1H), 0.92 (t, J = 8.3 Hz, 3H) ppm. ^{13}C NMR (125 MHz, $CDCl_3$): 159.8, 135.9, 131.1, 127.3, 116.8, 113.5, 101.7, 80.8, 72.7, 55.2, 42.6, 26.2, 9.35 ppm. IR υ_{max} (film): 3073, 2965, 2937, 2839, 1616, 1518, 1463, 1397, 1361, 1247, 1170, 1121, 1078, 1033, 995,992, 918, 828 cm^{-1}. HRMS calc. for

$C_{15}H_{20}O_3Na^+$: 271.1305. Found: 271.1295. Error (ppm): –3.75. The NMR NOE difference (NOEDIFF) experiment confirmed the 1,2-*syn* stereochemical relationship between C3 and C4.

(2*R*,3*R*)-3-((4-methoxybenzyl)oxy)-2-vinylpentan-1-ol (9)

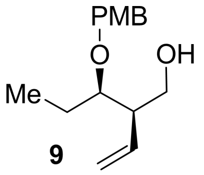

A solution of PMP acetal **18** (0.54 mmol, 132 mg) in CH_2Cl_2 (3 mL) was cooled to 0 °C. Then, DIBAL was added dropwise (1.35 mmol, 0.9 mL, 1.5 M solution in toluene) and the reaction was stirred for 45 min. The crude reaction was placed in a beaker containing HCl (7.8 mL, solution 0.5 M) and CH_2Cl_2 (7.8 mL) and the reaction mixture was stirred for 30 min at room temperature. The phases were separated and the aqueous layer was washed with CH_2Cl_2 (3 x 20 mL). The combined organic layers were washed with brine (2 x 20 mL) and dried over $MgSO_4$, filtered, and concentrated *in vacuum*. The crude reaction product was obtained as colorless oil (133 mg, 99%) and was used in the following step without further purification. R*f* 0.16 (10% EtOAc/hexanes); $[\alpha]^{20}_D$ +2.0 (c 1.3, $CHCl_3$). ^{1}H NMR (250 MHz, $CDCl_3$): 7.27 (d, *J* = 8.5 Hz, 2H), 6.88 (d, *J* = 8.5 Hz, 2H), 5.79 (ddd, J_1 = 17.3 Hz, J_2 = 10.4 Hz, J_3 = 8.7 Hz, 1H), 5.22–5.10 (m, 2H), 4.50 (q, *J* = 9.5 Hz, 2H), 3.81 (s, 3H), 3.76–3.63 (m, 2H), 3.15 (td, J_1 = 8.3 Hz, J_2 = 3.6 Hz, 1H), 2.60–2.50 (m, 1H), 1.75–1.45 (m, 2H), 0.91 (t, *J* = 3.9 Hz, 3H) ppm. ^{13}C NMR (100 MHz, $CDCl_3$): 159.2, 135.5, 130.5, 129.4, 118.3, 113.8, 81.7, 71.4, 63.9, 55.2, 48.5, 23.7, 10.2 ppm. IR υ_{max} (film): 3414, 2962, 2934, 2876, 1613, 1514, 1247, 1036, 917, 822 cm^{-1}.

HRMS calc. for $C_{15}H_{22}O_3Na^+$: 273.1461. Found: 273.1456. Error (ppm): –1.74.

(2*R*,3*R*)-3-((4-methoxybenzyl)oxy)-2-vinylpentyl 4-methylbenzenesulfonate (19)

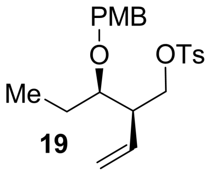

To a solution of alcohol **9** (0.37 mmol, 0.092 g) in CH_2Cl_2 (5 mL) were added triethylamine (1.10 mmol, 0.16 mL), DMAP (2 mol%, 0.001 g) and tosyl chloride (0.74 mmol, 0.14 g). The reaction was stirred for 4 h at room temperature. The reaction was quenched with distilled water (3 mL) and the organic layer was extracted with CH_2Cl_2 (3 x 25 mL). Then, the combined organic layers were washed with HCl (3 x 10 mL, 0.1 M solution) and brine (2 x 15 mL), dried over $MgSO_4$, filtered and concentrated *in vacuum*. The crude reaction product was purified by flash column chromatography (silica gel, EtOAc/hexanes 10/90) to give 134 mg of compound **19** (90%) as colorless oil. R*f* = 0.65 (30% EtOAc in hexanes). $[\alpha]^{20}_D$ –8.0 (c 1.75, $CHCl_3$). ^{1}H NMR (600 MHz, $CDCl_3$): 7.76 (d, *J* = 8.5 Hz, 2H), 7.30 (d, 8.5 Hz, 2H), 7.14 (d, *J* = 8.5 Hz, 2H), 6.83 (d, *J* = 8.5 Hz, 2H), 5.63 (ddd, J_1 = 17.2 Hz, J_2 = 10.9 Hz, J_3 = 9.0 Hz, 1H), 5.15 (dd, J_1 = 10.5 Hz, J_2 = 1.5 Hz, 1H), 5.10 (dt, J_1 = 17.2 Hz, J_2 = 1.5 Hz, 1H), 4.43 (d, *J* = 9.7 Hz, 1H), 4.26 (d, *J* = 11.0 Hz, 1H), 4.13 (dd, J_1 = 9.7 Hz, J_2 = 8.0 Hz, 1H), 3.96 (J_1 = 9.7 Hz, J_2 = 6.5 Hz, 1H), 3.8 (s, 3H), 3.40 (td, J_1 = 8.5 Hz, J_2 = 3.2 Hz, 1H), 2.62–2.57 (m, 1H), 2.41 (s, 3H), 1.62–1.55 (m, 1H), 1.44–1.36 (m, 1H), 0.83 (t, *J* = 7.2 Hz, 3H) ppm. ^{13}C NMR (150 MHz, $CDCl_3$): 159.0, 144.7, 133.2, 133.0, 130.6, 129.8, 129.1, 127.9, 119.5, 113.6, 78.9, 71.7, 70.4, 55.2, 46.4, 24.1, 21.6, 10.0 ppm. IR υ_{max} (film): 3554, 2963, 2926, 2878, 1597, 1457, 1353, 1172, 943, 812, 663, 551 cm^{-1}.

HRMS calc. for $C_{22}H_{28}O_5SNa^+$: 427.1550. Found: 427.1538. Error (ppm): -2.73.

1-methoxy-4-((((3*R*,4*S*)-4-methylhex-5-en-3-yl)oxy)methyl)benzene (10)

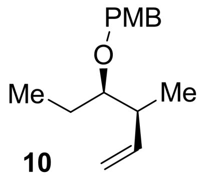
10

A solution of the tosylate **19** (0.25 mmol, 0.1 g) in THF (15 mL) was cooled to –78 °C. Then, $LiBH_4$ (1.23 mmol, 0.62 mL, 2M solution in THF) was added dropwise and the reaction was warmed to room temperature and stirred for 12 h. The reaction was cooled to 0 °C, distilled water (5 mL) was added carefully and the solvent was removed *in vacuum*. The organic layer was extracted with CH_2Cl_2 (3 x 20 mL). The combined organic layers were washed with HCl (3 x 10 mL, 0.1 M solution) and brine (2 x 15 mL), dried over $MgSO_4$, filtered and concentrated *in vacuum*. The crude reaction product was purified by flash column chromatography (silica gel, EtOAc/hexanes 5/95) to give 49 mg of compound **10** (85%) as colorless oil. R*f* = 0.8 (10% EtOAc in hexanes). $[\alpha]^{20}_{D}$ –27.0 (c 0.31, $CHCl_3$). ^{1}H NMR (500 MHz, $CDCl_3$): 7.27 (d, J = 8.5 Hz, 2H), 6.87 (d, J = 8.5 Hz, 2H), 5.83 (ddd, J_1 = 17.3 Hz, J_2 = 10.5 Hz, J_3 = 7.4 Hz, 1H), 5.04–5.01 (m, 2H), 4.46 (q, J = 9.7 Hz, 2H), 3.79 (s, 3H), 3.20–3.16 (m, 1H), 2.49 (br. s, J = 6.9 Hz, 1H), 1.53–1.45 (m, 2H), 1.02 (d, J = 7.4 Hz, 3H), 0.91 (t, J = 7.7 Hz, 3H) ppm. ^{13}C NMR (62.5 MHz, $CDCl_3$): 159.0, 141.1, 131.2, 129.2, 114.2, 113.6, 83.9, 71.4, 55.2, 40.2, 23.4, 15.1, 10.2 ppm. IR υ_{max} (film): 3406, 2965, 2934, 2818, 1613, 1514, 1463, 1249, 1174, 1069, 1035, 821 cm^{-1}.

HRMS calc. for $C_{15}H_{22}O_2H^+$: 235.1693. Found: 235.1682. Error (ppm): –4.49.

(2*E*,4*E*,6*S*,7*R*)-ethyl 7-((4-methoxybenzyl)oxy)-6-methylnona-2,4-dienoate (11)

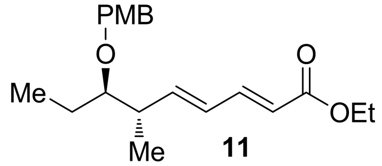
11

To a solution of olefin **10** (1.2 mmol, 0.282 g) in THF/acetone/buffer pH 7 (9.6 mL/9.6 mL/9.6 mL) were added OsO_4 (0.12 mmol, 0.6 mL, 2M solution in *t*-butanol) and NMO (1.56 mmol, 0.182 g). The reaction media was stirred for 30 min and after this time the volatile solvents were removed under reduced pressure. The crude reaction was diluted with EtOAc and washed with a saturated aqueous solution of sodium bisulfide (3 x 15 mL). The organic layer was extracted with EtOAc (3 x 30 mL), dried over $MgSO_4$, filtered and concentrated under reduced pressure. The crude reaction was purified by flash column chromatography (silica gel, EtOAc/hexanes 50/50). R*f* 0.6 (80% AcOEt/hexanes). The next step was the oxidative cleavage: to a solution of obtained the diol **20** (1.03 mmol, 0.276 g) in THF (40 mL) were added a buffer solution pH 7 (8 mL) and $NaIO_4$ (2.06 mmol, 0.45 g). The reaction media was stirred for 1 h at room temperature. Then, the reaction was diluted with EtOAc (30 mL), washed with saturated aqueous solution of sodium bisulfide (3 x 15 mL). The organic layer was extracted with EtOAc (3 x 30 mL). The combined organic layers were dried over $MgSO_4$ and concentrated under reduce pressure. The crude reaction product was obtained as colorless oil (243 mg, 86% for 2 steps) and was used in the following step without further purification. The last step was the HWE olefination: A solution of the (*E*)-ethyl 4-(diethoxyphosphoryl)but-2-enoate (2.66 mmol, 0.59 mL) in THF (14 mL) was cooled to –78 °C. Then, a solution of LiHMDS (2.33 mmol, 2.33 mL, 1M solution in THF) was added dropwise. The reaction was warmed to –25 °C and stirred for 30 min. After this time, the reaction was cooled to –78 °C and a solution of the aldehyde (1.0 mmol, 0.22 g in 5 mL of THF) was added dropwise. Then, the reaction was warmed to –25

°C and was stirred for 2 h at this temperature. The reaction was quenched by adding a saturated aqueous solution of NH_4Cl (10 mL). The organic layer was extracted with EtOAc (3 x 30 mL), dried over $MgSO_4$, filtered and concentrated under reduced pressure. The crude reaction was purified by flash column chromatography (silica gel, EtOAc/hexanes 8/92), to give 278 mg of compound **11** (84%) as a yellow oil. Rf = 0.74 (15% EtOAc in hexanes). $[\alpha]^{20}_D$ +5.0 (c 1.0, $CHCl_3$). ^{1}H NMR (600 MHz, $CDCl_3$): 7.29–7.24 (m, 3H), 6.87 (d, J = 8.5 Hz, 2H), 6.18 (dd, J_1 = 15.3 Hz, J_2 = 10.5 Hz, 1H), 6.12 (dd, J_1 = 15.3 Hz, J_2 = 7.5 Hz, 1H), 5.80 (d, J = 15.3 Hz, 1H), 4.45 (q, J = 11.2 Hz, 2H), 4.20 (q, J = 7.0 Hz, 2H), 3.80 (s, 3H), 3.22–3.19 (m, 1H), 2.58 (br. s, J = 6.9 Hz, 1H), 1.53–1.44 (m, 2H), 1.29 (t, J = 7.2 Hz, 3H), 1.05 (d, J = 6.8 Hz, 3H), 0.91 (t, J = 7.0 Hz, 3H) ppm. ^{13}C NMR (150 MHz, $CDCl_3$): 167.2, 159.0, 146.6, 145.0, 130.8, 129.3, 128.1, 119.5, 113.8, 83.6, 71.6, 60.1, 55.2, 39.9, 23.8, 15.5, 14.2, 9.9 ppm. IR υ_{max} (film): 2965, 2935, 2874, 1711, 1641, 1464, 1248, 1142, 1036, 822 cm^{-1}.

HRMS calc. for $C_{20}H_{28}O_4Na^+$: 355.1880. Found: 355.1869. Error (ppm): -2.99.

(2*E*,4*E*,6*S*,7*R*)-7-((4-methoxybenzyl)oxy)-6-methylnona-2,4-dienoic acid (22)

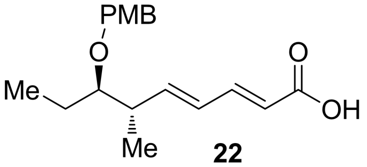

22

A solution of ester **11** (0.54 mmol, 0.17 g) in EtOH (18 mL) was cooled to 0 °C and were added H_2O (9 mL) and an aqueous solution of KOH (2.16 mL, 10%). The reaction was warmed to room temperature and stirred for 36 h. The reaction was quenched by adding a saturated aqueous solution of citric acid (until pH 4, 5 mL) and was concentrated *in vacuum*. After, the organic layer was extracted with EtOAc (5 x 30 mL), dried over $MgSO_4$, filtered and concentrated under reduced pressure. The crude reaction product was obtained as colorless oil the pure form (162 mg, 99%) and was used in the following step without purification. R*f* = 0.76 (EtOAc). $[\alpha]^{20}_D$ +10.0 (c 0.91, MeOH). ^{1}H NMR (250 MHz, $CDCl_3$): 7.32–7.16 (m, 3H), 6.80 (d, J = 8.5 Hz, 2H), 6.20–6.04 (m, 2H), 5.73 (d, J = 15.7 Hz, 1H), 4.39 (q, J = 11.5 Hz, 2H), 3.73 (s, 3H), 3.14 (q, J = 6.4 Hz, 1H), 2.55–2.48 (m, 1H), 1.54–1.34 (m, 2H), 0.99 (d, J = 7.0 Hz, 3H), 0.85 (t, J = 7.0 Hz, 3H) ppm. ^{13}C NMR (125 MHz, $CDCl_3$): 159.1, 148.2, 147.4, 130.7, 129.3, 128.0, 118.5, 118.4, 113.7, 83.4, 71.6, 55.2, 40.1, 23.8, 15.5, 9.8 ppm. IR υ_{max} (film): 2965, 2935, 2875, 2837, 1686, 1636, 1614, 1514, 1462, 1419, 1303, 1248, 1003, 821 cm^{-1}.

HRMS calc. for $C_{18}H_{24}O_4Na^+$: 327.1567. Found: 327.1556. Error (ppm): –3.21.

(2*E*,4*E*,6*S*,7*R*)-*S*-(2-acetamidoethyl) 7-hydroxy-6-methylnona-2,4-dienethioate (2b)

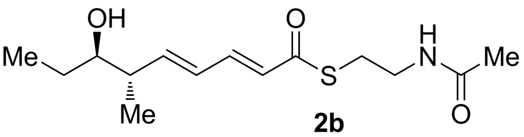

2b

Step 1: To a solution of the acid **22** (0.25 mmol, 0.071 g) in dry DMF (2.5 mL) at room temperature were added HOBt (0.3 mmol, 0.4 g) and *N*-(2-mercaptoethyl)acetamide (1.25 mmol, 0.13 g). Then, the reaction was cooled to 0 °C and a solution of DCC (0.3 mmol, 0.062 g) in dry DMF (1 mL) was added. The reaction was warmed to room temperature and stirred for 48 h. After this time, the reaction was quenched by the addition of saturated aqueous NH_4Cl solution (5 mL). The aqueous layer was extracted with EtOAc (3 x 20 mL). The combined organic layers were dried over $MgSO_4$, filtered and concentrated *in vacuum*. The crude mixture was purified

by flash column chromatography (silica gel and hexanes/EtOAc, 70:30). *Rf* = 0.76 (EtOAc). We verified the presence of 1,3-dicyclohexylurea in the purified product **23**. Thus, the mixture of compounds was used in the following step. Step 2: To a stirring solution of the obtained mixture in CH_2Cl_2 (2.8 mL) and pH 7.0 buffer (0.3 mL) at 0 °C, and DDQ (0.19 mmol, 42.6 mg) was added. The resulting mixture was stirred at room temperature for 30 min. Distilled H_2O (0.9 mL) was added, the mixture was filtered and the supernatant was collected. The aqueous layer was extracted with CH_2Cl_2 (5 x 20 mL). The combined organic extracts were dried with anhydrous Na_2SO_4 and concentrated on a rotary evaporator. The crude mixture was purified by flash column chromatography (silica gel and hexanes/EtOAc, 95:5) to give the product (0.39 mg, 55% for 2 steps) as colorless oil. R*f* = 0.39 (EtOAc). $[\alpha]^{20}_{D}$ –28.0 (c 1.01, MeOH). ^{1}H NMR (600 MHz, $CDCl_3$): 7.24 (dd, J_1 = 15.3 Hz, J_2 = 9.9 Hz, 1H), 6.26–6.18 (m, 2H), 6.14 (d, J = 15.3 Hz, 1H), 5.92 (br. s, 1H), 3.49–3.43 (m, 3H), 3.11 (t, J = 6.5 Hz, 2H), 2.42–2.37 (m, 1H), 1.96 (s, 3H), 1.58–1.49 (m, 2H), 1.46–1.38 (m, 1H), 1.10 (t, J = 7.0 Hz, 3H), 0.97 (t, J = 7.3 Hz, 3H) ppm. ^{13}C NMR (150 MHz, $CDCl_3$): 190.4, 170.3, 147.9, 141.5, 128.8, 126.6, 76.4, 43.0, 39.9, 28.3, 27.5, 23.2, 16.4, 10.1 ppm. IR υ_{max} (film): 3289, 2964, 2932, 2876, 1655, 1634, 1597, 1553, 1291, 1025, 999, 819 cm^{-1}.

HRMS calc. for $C_{14}H_{23}NO_3SH^+$: 286.1471. Found: 286.1464. Error (ppm): –2.45.

3.2. Pentaketide 3b.

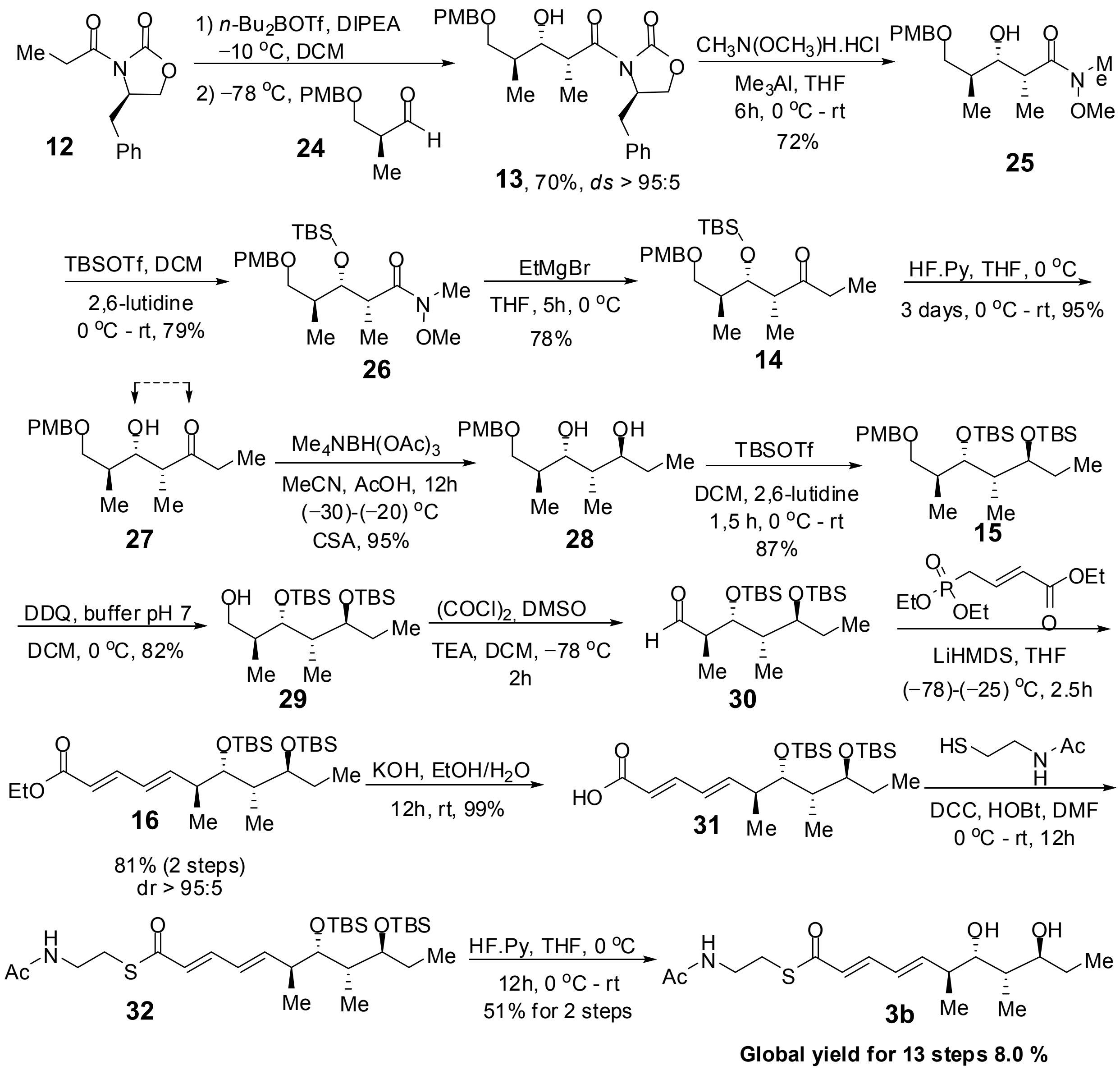

Scheme S2. Substrate **3b** synthesis.

(*R*)-4-benzyl-3-((2*R*,3*S*,4*S*)-3-hydroxy-5-((4-methoxybenzyl)oxy)-2,4-dimethylpentanoyl)oxazolidin-2-one (13)[11]

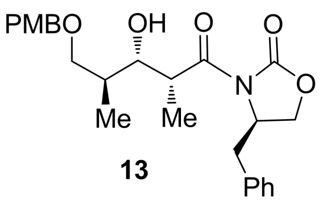

Freshly distilled *n*Bu2BOTf (14.52 mmol, 3.7 mL, d = 1.089 g/mL) was slowly added to a solution of chiral auxiliary (*R*)-4-benzyl-3-propionyloxazolidin-2-one (**12**) (9.68 mmol, 2.26 g) in CH_2Cl_2 (18.3 mL) at −10 °C under an argon atmosphere. Then, DIPEA (16.5 mmol, 3 mL) was added dropwise. The temperature was reduced to −78 °C and a cooled solution (ice bath) of (*S*)-3-((4-methoxybenzyl)oxy)-2-methylpropanal (12.6 mmol, 2.63 g) in CH_2Cl_2 (13 mL) was added dropwise. The reaction mixture was maintained at −78 °C for 30 min and then at −10 °C for 2 h. Then, phosphate buffer (22 mL) and methanol (62 mL) were added, followed by the slow addition of a 2:1 mixture of MeOH (57 mL)

and H_2O_2 (25% aq.; 28 mL), with stirring, over 1 h at –5 °C. The solvents were removed under vacuum and the crude mixture was extracted with ethyl ether (3 x 30 mL). The organic layer was washed with $NaHCO_3$ (saturated aq.; 120 mL) and NaCl (saturated aq.; 120 mL), dried with anhydrous Na_2SO_4, and the solvents were evaporated under vacuum. The crude reaction product was purified by flash column chromatography (silica gel, EtOAc/hexanes 15/85) to give 2.49 g of compound **13** (70%, dr = > 95:5) as a pale yellow oil. R*f* = 0.32 (35% EtOAc in hexanes). $[\alpha]^{20}_{D}$ –17,1 ° (c 1.1, $CHCl_3$). ^{1}H NMR (250 MHz, $CDCl_3$): 7.35–7.18 (m, 7H), 6.85 (d, *J* = 8.5 Hz, 2H), 4.69–4.60 (m, 1H), 4.42 (s, 2H), 4.13 (d, *J* = 4.7 Hz, 2H), 3.98–3.83 (m, 2H), 3.77 (s, 3H), 3.74–3.73 (m, 1H), 3.57–3.52 (m, 2H), 3.29 (dd, J_1 = 13.5 Hz, J_2 = 3.0 Hz, 1H), 2.76 (dd, J_1 = 13.5 Hz, J_2 = 9.5 Hz, 1H), 2.03–1.93 (m, 1H), 1.25 (d, *J* = 7.0 Hz, 3H), 0.94 (d, *J* = 7.0 Hz, 3H) ppm. ^{13}C NMR (62.5 MHz, $CDCl_3$): 176.0, 159.1, 153.0, 135.2, 129.7, 129.3, 129.2, 128.8, 127.2, 113.7, 75.2, 74.4, 73.0, 66.0, 55.4, 55.1, 40.5, 37.6, 35.8, 13.4, 9.6 ppm.

(2*R*,3*S*,4*S*)-3-hydroxy-*N*-methoxy-5-((4-methoxybenzyl)oxy)-*N*,2,4-trimethylpentanamide (25)[11]

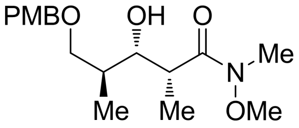

25

Me_3Al (2.0 m in toluene, 4.63 mmol, 2.31 mL) was added slowly to a suspension of *N*,*O*-dimethylhydroxyamine (4,63 mmol, 452 mg) in THF (3.37 mL) cooled to 0 ºC. The reaction mixture was maintained at 0 °C for 30 min and then at room temperature for 90 min. Next, the reaction temperature was adjusted to –20 °C, and a solution of aldol product **13** (1.16 mmol, 510 mg) in THF (2.5 mL) was added by cannula. The temperature was increased to 0 °C and the reaction mixture was stirred for 90 min. Then, the reaction mixture was transferred by cannula to a flask containing HCl (1.0 M; 9 mL) and CH_2Cl_2 (9 mL) and the resulting mixture was stirred at 0 °C for 90 min. The aqueous layer was extracted with CH_2Cl_2 (3 x 25 mL), and the organic layer was washed with NaCl (saturated aq.; 25 mL) and dried with anhydrous Na_2SO_4. The solvents were removed under vacuum. The crude mixture was diluted with diethyl ether (5 mL) and hexanes (5 mL) and cooled to 5 ºC for crystallizing the chiral auxiliary. Thus, the chiral auxiliary was removed by filtration and the crude reaction product was purified by flash column chromatography (silica gel, EtOAc/hexanes 20/80) to give 271 mg of compound **25** (72%) as yellow oil. **Rf** = 0.39 (70% EtOAc in hexanes). $[\alpha]^{20}_{D}$ –12.5 ° (c 3.4 $CHCl_3$). ^{1}H NMR (500 MHz, $CDCl_3$): 7.25 (d, *J* = 8.5 Hz, 2H), 6.86 (d, *J* = 8.5 Hz, 2H), 4.44 (q, *J* = 11.5 Hz, 2H), 4.03 (d, *J* = 3.0 Hz, 1H), 3.77 (s, 3H), 3.64 (dt, J_1 = 8.0 Hz, J_2 = 3.2 Hz, 1H), 3.77 (s, 3H), 3.63–3.60 (m, 1H), 3.53 (dd, J_1 = 9.0 Hz, J_2 = 6.0 Hz, 1H), 3.16 (s, 3H), 3.04 (br. s, 1H), 1.89–1.83 (m, 1H), 1.17 (d, *J* = 7.0 Hz, 3H), 0.99 (d, *J* = 7.0 Hz, 3H) ppm. ^{13}C NMR (125 MHz, $CDCl_3$): 177.6, 258.8, 130.3, 128.9, 113.4, 77.2, 73.7, 72.6, 72.4, 61.1, 54.6, 36.3, 35.7, 14.0, 10.3 ppm.

(2*R*,3*S*,4*S*)-3-((*tert*-butyldimethylsilyl)oxy)-*N*-methoxy-5-((4-methoxybenzyl)oxy)-*N*,2,4-trimethylpentanamide (26)[11]

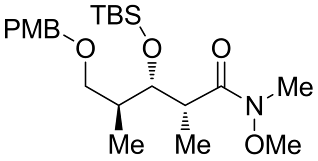

26

CH_2Cl_2 (1,74 mL) was added to a flask containing the Weinreb amide **25** (1.43 mmol, 464 mg), and the resulting solution was cooled to 0 °C. Then, 2,6-lutidine (1.86 mmol, 0.21 mL) was added, followed by TBSOTf (1.71 mmol, 0.39 mL). The reaction mixture was stirred at 0 °C for 10 min and then at 25 °C

for 1.5 h. After this time, ethyl ether (10 mL) and cold $NaHSO_4$ (1 M; 5 mL) were added. The reaction mixture was extracted with ethyl ether (3 x 15 mL), and the organic extracts were washed with distilled water (10 mL), $NaHCO_3$ (saturated aq.; 15 mL), and NaCl (saturated aq.; 15 mL). The combined organic extracts were dried with anhydrous Na_2SO_4 and concentrated under reduced pressure. The crude reaction product was purified by flash column chromatography (silica gel, hexanes/EtOAc 85/15), providing 495 mg of product **26** as a pale yellow oil in 79%. R*f* = 0.5 (20% EtOAc in hexanes). $[\alpha]^{20}_{D}$ –10.0 (c 1.2, $CHCl_3$). ^{1}H NMR (500 MHz, $CDCl_3$): 7.21 (d, *J* = 8.5 Hz, 2H), 6.83 (d, *J* = 8.5 Hz, 2H), 4.36 (d, *J* = 4.3 Hz, 2H), 3.92 (dd, J_1 = 8.3 Hz, J_2 = 3.0 Hz, 1H), 3.76 (s, 3H), 3.55–3.52 (m, 4H), 3.13 (dd, J_1 = 9.2 Hz, J_2 = 8.0 Hz, 2H), 3.08 (s, 3H), 1.94–1.90 (m, 1H), 1.09 (d, *J* = 6.3 Hz, 3H), 0.98 (d, *J* = 7.0 Hz, 3H), 0.87 (s, 9H), 0.04 (s, 6H) ppm. ^{13}C NMR (125 MHz, $CDCl_3$): 176.5, 158.9, 130.7, 129.1, 113.5, 75.9, 72.5, 71.7, 61.0, 55.1, 39.1, 38.7, 31.9, 26.0, 18.2, 15.2, 14.8, –3.9, –4.0 ppm.

(4*R*,5*S*,6*S*)-5-((*tert*-butyldimethylsilyl)oxy)-7-((4-methoxybenzyl)oxy)-4,6-dimethylheptan-3-one (14)[11]

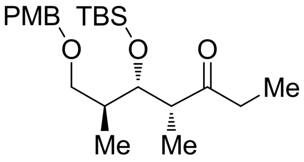

14

EtMgBr (2.0 M in THF; 10.4 mmol, 5.2 mL) was added dropwise to a solution of amide **26** (2,7 mmol, 1.1 g) in dry THF (45 mL) at 0 °C. The reaction was complete after 1.5 h, and it was quenched by the careful addition of NH_4Cl (saturated aq., 55 mL). The phases were separated and the aqueous layer was extracted with ethyl ether (3 x 100 mL). The combined organic layers were dried with Na_2SO_4 and concentrated under reduced pressure. Purification by flash column chromatography (silica gel and hexane/EtOAc, 80:20) gave product **14** (861 mg, 78%) as yellow oil. R*f* = 0.5 (20% EtOAc in hexanes). $[\alpha]^{20}_{D}$ –12.0 (c 2.2 $CHCl_3$). ^{1}H NMR (600 MHz, $CDCl_3$): 7.23 (d, *J* = 8.5 Hz, 2H), 6.86 (d, *J* = 8.5 Hz, 2H), 4.37 (d, *J* = 4.5 Hz, 2H), 3.99 (dd, J_1 = 5.6 Hz, J_2 = 4.6 Hz, 1H), 3.79 (s, 3H), 3.47 (dd, J_1 = 9.0 Hz, J_2 = 5.8 Hz, 1H), 3.17 (dd, J_1 = 9.0 Hz, J_2 = 6.5 Hz, 1H), 2.80–2.75 (m, 1H), 2.48–2.37 (m, 2H), 1.87–2.75 (m, 1H), 1.06 (d, J_1 = 7.0 Hz, 3H), 0.97 (t, *J* = 7.0 Hz, 3H), 0.93 (d, *J* = 0.93, 3H), 0.86 (s, 9H), 0.03 (s, 3H), 0.01 (s, 3H) ppm. ^{13}C NMR (150 MHz, $CDCl_3$): 214.4, 159.0, 130.6, 129.1, 113.6, 74.0, 72.6, 71.7, 55.2, 49.5, 38.8, 35.0, 26.0, 18.3, 14.8, 13.4, 7.5, –3.8, –4.2 ppm. IR υ_{max} (film): 2954, 2930, 2855, 1709, 1612, 1513, 1459, 1247, 1092, 1036, 831, 773 cm^{-1}.

(4*R*,5*S*,6*S*)-5-hydroxy-7-((4-methoxybenzyl)oxy)-4,6-dimethylheptan-3-one (27)

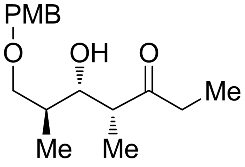

27

To a stirring solution of TBS ether **14** (0.11 mmol, 0.045 g) and THF (0.5 mL) in a polyethylene flask at 0 °C was added a solution of HF:pyridine (0.5 mL, 60-70% solution). The reaction was stirred for 3 days at room temperature and then portions of solid $NaHCO_3$ (500 mg) were added. The aqueous layer was extracted with EtOAc (3 x 15 mL), and the combined organic layers were dried over $MgSO_4$, filtered and concentrated. The crude product was purified by flash chromatography in silica gel (10% EtOAc/hexane) to give alcohol **27** (31 mg, 95%) as white solid. R*f* 0.43 (20% EtOAc/hexanes). $[\alpha]^{20}_{D}$ +11.0 (c 2.95 $CHCl_3$). M.p.: 63–65 °C. ^{1}H NMR (500 MHz, $CDCl_3$): 7.22 (d, *J* = 8.5 Hz, 2H), 6.86 (d, *J* = 8.5 Hz, 2H), 4.43 (d, *J* = 3.2 Hz, 2H), 3.86 (dt, J_1 = 8.1 Hz, J_2 = 3.2 Hz, 1H), 3.79 (s, 3H), 3.56–3.50 (m, 3H), 2.63–2.58 (m, 1H),

2.51 (q, *J* = 7.0 Hz, 2H), 1.90–1.82 (m, 1H), 1.11 (d, *J* = 7.0 Hz, 3H), 1.03 (t, *J* = 7.3 Hz, 3H), 0.88 (d, *J* = 7.0 Hz, 3H) ppm. ^{13}C NMR (125 MHz, $CDCl_3$): 215.2, 159.2, 129.9, 129.3, 113.7, 75.2, 74.2, 73.0, 55.2, 48.3, 35.8, 34.2, 13.8, 9.25, 7.68 ppm. IR υ_{max} (film): 3485, 2971, 2937, 2878, 1707, 1612, 1513, 1457, 1370, 1301, 1247, 1083, 1034, 974, 820 cm^{-1}.
HRMS calc. for $C_{17}H_{26}O_4Na^+$: 317.1723. Found: 317.1718. Error (ppm): -1.58.

(2*S*,3*S*,4*S*,5*S*)-1-((4-methoxybenzyl)oxy)-2,4-dimethylheptane-3,5-diol (28)

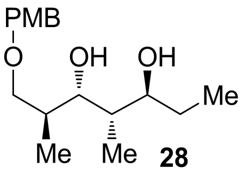

To a stirring suspension of $Me_4NBH(OAc)_3$ (0.894 mmol, 3.4 g) in MeCN (2.5 mL) at room temperature was added AcOH (2.5 mL). The mixture was stirred for 30 min and after this time was cooled to –40 ºC. Then, a solution of alcohol **28** (0.85 mml, 0,249 g) in MeCN (2.5 mL) was added dropwise. After, a solution of CSA (0.43 mmol, 100 mg) in MeCN (2.5 mL) and AcOH (2.5 mL) was added dropwise. The reaction mixture was warmed to –20 ºC and stirred for 20 h at this temperature. Then, the crude reaction was placed into a beaker containing saturated $NaHCO_3$ (350 mL), under stirring. After cessation of effervescence, a saturated aqueous solution of potassium and sodium tartrate (44 mL) and diethyl ether (44 mL) were added. The reaction mixture was stirred vigorously for 8 h. The phases were separated and the aqueous layer was extracted with ethyl ether (3 x 50 mL). The combined organic layers were dried with Na_2SO_4 and concentrated under reduced pressure. The reaction crude was used without purification in the next step. The product **28** was obtained as a colorless solid in 239 mg, 95% of yield. R*f* 0.48 (40% AcOEt/hexanes). $[\alpha]^{20}_D$ –8.0 (c 4.0 MeOH). M.p.: 57–59 ºC. ^{1}H NMR (500 MHz, $CDCl_3$): 7.24 (d, *J* = 8.5 Hz, 2H), 6.88 (d, *J* = 8.5 Hz, 2H), 4.45 (d, *J* = 3.0 Hz, 2H), 4.16 (s, 1H), 3.88 (d, *J* = 9.2 Hz, 1H), 3.81 (s, 3H), 3.58 (dd, J_1 = 9.2 Hz, J_2 = 4.1 Hz, 1H), 3.47 (t, *J* 9.2 Hz, 2H), 3.13 (d, *J* = 7.0 Hz, 1H), 2.02–1.97 (m, 1H), 1.63–1.55 (m, 3H), 1.01 (d, *J* = 7.2 Hz, 3H), 0.96 (t, *J* = 7.2 Hz, 3H), 0.74 (d, *J* = 7.0 Hz, 3H) ppm. ^{13}C NMR (125 MHz, $CDCl_3$): 159.3, 129.5, 129.3, 113.9, 77.5, 76.62, 76.60, 73.2, 55.2, 37.7, 35.6, 28.5, 12.9, 10.6, 10.4 ppm. IR υ_{max} (film): 3424, 2963, 2934, 1612, 1513, 1245, 1081, 1030, 969 cm^{-1}.
HRMS calc. for $C_{17}H_{28}O_4Na^+$: 319.1880. Found: 319.1880. Error (ppm): 0.

(4*S*,5*S*,6*S*)-4-ethyl-6-((*S*)-1-((4-methoxybenzyl)oxy)propan-2-yl)-2,2,5-trimethyl-1,3-dioxane (33)

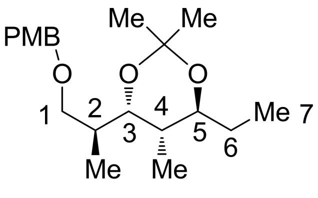

33

Compound **33** was synthetized to confirm the stereochemical relationship between C3 and C5. NMR analysis of acetonide **33** confirmed the 1,3-anti stereochemical relationship between C3 and C5.[12] The diol **28** (0.15 mmol, 0.044 g) was dissolved in 2,2-dimethoxypropane (2.4 ml), followed by addition of CSA (0,012 mmol, 3 mg) at room temperature. The reaction mixture was stirred for 18 h before it was diluted with EtOAc (2 mL) and aqueous solution of saturated aqueous $NaHCO_3$ (2 mL). The organic layer was separated and the aqueous layer was further extracted with EtOAc (3 x 10 mL). The combined organic layer was washed with brine (5 mL), dried over $MgSO_4$, filtered and concentrated. The crude product was purified by flash chromatography (10%

EtOAc/hexane) to afford the desired acetonide **33** (33 mg, 66% yield) as colorless oil. R*f* 0.75 (10% EtOAc/hexanes). ^{1}H NMR (250 MHz, $CDCl_3$): 7.25 (d, *J* = 8.5 Hz, 2H), 6.86 (d, *J* = 8.5 Hz, 2H), 4.41 (s, 2H), 3.80 (s, 3H), 3.64 (dd, J_1 = 11.0 Hz, J_2 = 4.1 Hz, 1H), 3.56 (dd, J_1 = 8.6 Hz, J_2 = 3.0 Hz, 1H), 3.38 (dd, J_1 = 8.6 Hz, J_2 = 6.2 Hz, 1H), 3.14 (td, J_1 = 9.7 Hz, J_2 = 4.4 Hz, 1H), 1.87–1.79 (m, 1H), 1.68–1.45 (m, 3H), 1.32 (s, 3H), 1.29 (s, 3H), 0.97–0.85 (m, 9H) ppm. ^{13}C NMR (62.5 MHz, $CDCl_3$): 158.9, 131.0, 129.0, 113.6, 100.5, 76.6, 72.7, 72.2, 69.9, 55.2, 37.8, 33.6, 27.7, 24.9, 23.7, 13.4, 11.7, 10.4 ppm. ^{13}C NMR DEPT 135 (62.5 MHz, $CDCl_3$): 129.0, 113.6, 76.7, 72.9, 72.2, 69.9, 55.2, 37.8, 33.6, 27.7, 24.9, 23.7, 13.4, 11.8, 10.4 ppm. IR υ_{max} (film): 2963, 2934, 2876, 1612, 1511, 1457, 1377, 1245, 1222, 1170, 1038, 1008, 989, 881 cm^{-1}.

HRMS calc. for $C_{20}H_{32}O_4Na^+$: 359.2193. Found: 359.2190. Error (ppm): –0.84.

(5S,6S,7S)-5-ethyl-7-((S)-1-((4-methoxybenzyl)oxy)propan-2-yl)-2,2,3,3,6,9,9,10,10-nonamethyl-4,8-dioxa-3,9-disilaundecane (15)

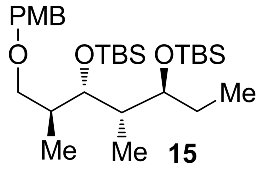

15

CH_2Cl_2 (22 mL) was added to a flask containing the diol **28** (1.5 mmol, 440 mg) and the resulting solution was cooled to 0 °C. Then, 2,6-lutidine (9.0 mmol, 1.05 mL) was added, followed by TBSOTf (4.5 mmol, 0.82 mL). The reaction mixture was stirred at 0 °C for 10 min and then at 25 °C for 1.5 h. After this time, ethyl ether (40 mL) and cold $NaHSO_4$ (1 M; 20 mL) were added. The reaction mixture was extracted with ethyl ether (3 x 30 mL) and the organic extracts were washed with distilled water (20 mL), $NaHCO_3$ (saturated aq. 30 mL), and NaCl (saturated aq., 30 mL). The combined organic extracts were dried with anhydrous Na_2SO_4 and concentrated under reduced pressure. The crude reaction product was purified by flash column chromatography (silica gel, hexanes/EtOAc 95/5), providing 683 mg (87%) of product **15** as a colorless oil. R*f* = 0.57 (10% EtOAc in hexanes). $[\alpha]^{20}_{D}$ –13.0 (c 2.6 $CHCl_3$). ^{1}H NMR (500 MHz, $CDCl_3$): 7.25 (d, *J* = 8.5 Hz, 2H), 6.87 (d, *J* = 8.5 Hz, 2H), 4.42 (d, *J* = 6.0 Hz, 2H), 3.80 (s, 3H), 3.66 (t, *J* = 4.0 Hz, 1H), 3.54–3.50 (m, 2H), 3.22 (t, *J* = 9.0 Hz, 1H), 1.98–1.91 (m, 1H), 1.79–1.72 (m, 1H), 1.45–1.36 (m, 2H), 0.97 (d, *J* = 7.0 Hz, 3H), 0.88–0.84 (m, 21H), 0.88 (d, *J* = 8.0 Hz, 3H), 0.04–0.03 (m, 12H) ppm. ^{13}C NMR (150 MHz, $CDCl_3$): 159.0, 130.8, 129.0, 113.7, 75.3, 74.8, 72.7, 72.3, 55.2, 44.1, 39.5, 26.1, 25.9, 24.8, 18.4, 18.1, 14.7, 10.7, 9.0, –3.6, –3.8, –4.1, –4.2 ppm. IR υ_{max} (film): 2956, 2930, 2855, 1513, 1248, 1040, 835, 771 cm^{-1}.

HRMS calc. for $C_{29}H_{56}O_4Si_2H^+$: 525.3790. Found: 525.3791. Error (ppm): 0.19.

(2S,3S,4S,5S)-3,5-bis((*tert*-butyldimethylsilyl)oxy)-2,4-dimethylheptan-1-ol (29)

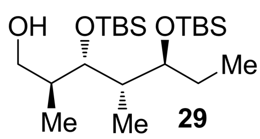

29

The compound **15** (1.0 mmol, 521 mg) was dissolved in a mixture of CH_2Cl_2 (19 mL) and pH 7.0 buffer (2.42 mL) at 0 °C, and DDQ (1.21 mmol, 274 mg) was added. The resulting mixture was stirred at room temperature for 30 min. Distilled H_2O (1.9 mL) was added, the mixture was filtered, and the supernatant was collected. The aqueous layer was extracted with CH_2Cl_2 (5 x 20 mL). The combined organic extracts were dried with anhydrous Na_2SO_4 and

concentrated on a rotary evaporator. The crude mixture was purified by flash column chromatography (silica gel and hexanes/EtOAc, 95:5) to give 331 mg (82%) of the desired product as colorless oil. R*f* = 0.39 (10% EtOAc in hexanes). $[\alpha]^{20}_D$ −1.1 (c 4.2 $CHCl_3$). ^1H NMR (250 MHz, $CDCl_3$): 3.82–3.73 (m, 2H), 3.58–3.52 (m, 2H), 2.60 (br. s, 1H), 1.90–1.72 (m, 2H), 1.54–1.35 (m, 2H), 1.0 (d, *J* = 7.0 Hz, 3H), 0.90–0.82 (m, 24H), 0.09–0.06 (m, 12H) ppm. ^{13}C NMR (125 MHz, $CDCl_3$): 76.5, 75.2, 65.3, 40.9, 40.2, 26.0, 25.9, 25.1, 18.3, 18.1, 14.5, 11.3, 8.0, −3.5, −3.8, −4.1, −42 ppm. IR υ_{max} (film): 1472, 1463, 1252, 1056, 1004, 833, 771, 674 cm^{-1}.

HRMS calc. for $C_{21}H_{48}O_3Si_2H^+$: 405.3215. Found: 405.3208. Error (ppm): −1.73.

(2E,4E,6S,7S,8S,9S)-ethyl 7,9-bis((*tert*-butyldimethylsilyl)oxy)-6,8 -dimethylundeca-2,4-dienoate (16)

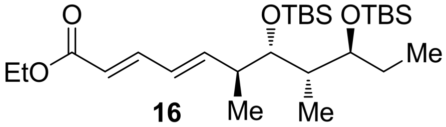

16

Step 1: DMSO (2.3 mmol, 0.17 mL) was added to a solution of oxalyl chloride (1.84 mmol, 0.16 mL) in CH_2Cl_2 (7 mL) at −78 °C. After 30 min, alcohol **29** (1.14 mmol, 460 mg) dissolved in CH_2Cl_2 (3.5 mL) was added. After stirring for 30 min at −78 °C, triethylamine (8.4 mmol, 1.16 mL) was added and the resulting mixture was stirred for 1.5 h at −78 °C. After this time, the reaction was quenched by adding NH_4Cl (saturated aq., 6 mL). The phases were separated and the aqueous layer was extracted with ethyl ether (3 x 25 mL). The combined organic layers were dried with anhydrous Na_2SO_4, filtered, and concentrated on a rotary evaporator. The crude reaction product was used in the following step without further purification. Rf = 0.58 (5% EtOAc in hexanes). Step 2: To a solution of (2.59 mmol, 0.56 mL) in dry THF (15.6 mL) at −78 °C was added LiHMDS (2.26 mmol, 2.26 mL, 1M in THF). The reaction was warmed to −25 °C and stirred for 30 min. Then, the mixture reaction was cooled to −78 °C and the aldehyde **30** (0.98 mmol, 0.394 g) was added as a solution in THF (5 mL) dropwise. After, the reaction mixture was warmed to −25 °C and stirred for 2 h at this temperature. The reaction was quenched by the addition of saturated aqueous NH_4Cl solution (10 mL). The aqueous layer was extracted with EtOAc (3 x 20 mL). The combined organic layers were dried over $MgSO_4$, filtered and concentrated *in vacuum*. The crude product was purified by flash chromatography on silica gel (8% EtOAc/hexane) to give the desired product (0.458 g, 81%, 2 steps) as yellow oil. R*f* 0.76 (10% EtOAc/hexanes). $[\alpha]^{20}_D$ −13.0 ° (c 1.7 $CHCl_3$). ^1H NMR (500 MHz, $CDCl_3$): 7.30–7.27 (m, 1H), 6.22–6.20 (m, 2H), 5.83 (d, *J* = 16.0 Hz, 1H), 4.24 (q, *J* = 7.0 Hz, 2H), 3.66 (dd, J_1 = 5.4 Hz, J_2 = 3.2 Hz, 1H), 3.54 (td, J_1 = 8.2 Hz, J_2 = 3.2 Hz, 1H), 2.47–2.41 (m, 1H), 1.74–1.67 (m, 1H), 1.46–1.42 (m, 1H), 1.33 (t, *J* = 7.0 Hz, 3H), 1.09 (d, *J* = 7.0 Hz, 3H), 0.94 (s, 9H), 0.91–0.87 (m, 12H), 0.83 (d, *J* = 7.0 Hz, 3H), 0.09 (s, 6H), 0.06 (s, 3H), 0.04 (s, 4H) ppm. ^{13}C NMR (125 MHz, $CDCl_3$): 167.2, 146.4, 145.0, 128.2, 119.5, 76.3, 74.6, 60.1, 43.3, 42.0, 26.1, 25.8, 24.7, 18.4, 18.1, 17.1, 14.3, 10.9, 9.0, −3.4, −3.7, −4.2, −4.2 ppm. IR υ_{max} (film): 2960, 2930, 2855, 1716, 1644, 1256, 1002, 835, 771 cm^{-1}.

HRMS calc. for $C_{27}H_{54}O_4Si_2H^+$: 499.3633 Found: 499.3641.Error (ppm): 1.60.

(2*E*,4*E*,6*S*,7*S*,8*S*,9*S*)-7,9-*bis*((*tert*-butyldimethylsilyl)oxy)-6,8-dimethylundeca-2,4-dienoic acid (31)

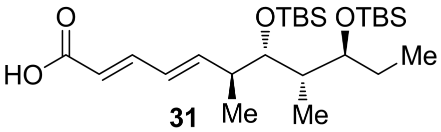

31

To a solution of the ester **16** (0.15 mmol, 0.073 g) in EtOH (5.1 mL) at 0 ºC were added H_2O (2.55 mL) and an aqueous solution of KOH 10% (0.6 mL). The reaction was warmed to room temperature and stirred for 36 h. After this time, an aqueous solution of citric acid was added until pH 4 (2 mL). The reaction crude was concentrated in *vacuum* and diluted with EtOAc (10 mL). The organic layer was extracted with EtOAc (3 x 15 mL). The combined organic layers were dried over $MgSO_4$, filtered and concentrated *in vacuum*. The crude reaction product was used in the following step without further purification. The corresponding acid was isolated as a white solid (70 mg, 99%). R*f* 0.1 (3% MeOH/EtOAc). $[\alpha]^{20}_D$ –17.0 (c 2.52 MeOH). M.p.= 88–89 ºC. ^{1}H NMR (500 MHz, MeOD): 7.23 (dd, J_1 = 15.4 Hz; J_2 = 10.5 Hz, 1H), 6.28 (dd, J_1 = 15.4 Hz, J_2 = 10.5 Hz, 1H), 6.20 (dd, J_1 = 7.5 Hz, J_2 = 15.4 Hz, 1H), 5.79 (d, J = 15.4 Hz, 1H), 3.72 (dd, J_1 = 5.0 Hz, J_2 = 3.3 Hz, 1H), 3.59–3.56 (m, 1H), 2.51–2.45 (m, 1H), 1.77–1.70 (m, 1H), 1.43–1.41 (m, 2H), 1.09 (d, J_1 = 7.0 Hz, 3H), 0.93 (s, 9H), 0.89–0.86 (m, 12H), 0.85 (d, J_1 = 7.0 Hz, 3H), 0.09 (s, 6H), 0.05–0.04 (m, 6H) ppm. ^{13}C NMR (125 MHz, MeOD): 170.5, 147.4, 146.7, 129.7, 121.0. 77.5, 76.1, 44.6, 43.2, 26.7, 26.5, 25.7, 19.3, 19.0, 17.1, 11.5, 9.7, –3.1, –3.2, –3.7, –3.8 ppm. IR υ_{max} (film): 2956, 2930, 2857, 1690, 1636, 1616, 1468, 1254, 1002, 855, 771 cm^{-1}.

HRMS calc. for $C_{25}H_{50}O_4Si_2H^+$: 471.3320. Found: 471.3316. Error (ppm): –0.85.

(2*E*,4*E*,6*S*,7*S*,8*S*,9*S*)-S-(2-acetamidoethyl) 7,9-dihydroxy-6,8-dimethylundeca-2,4-dienethioate (3b)

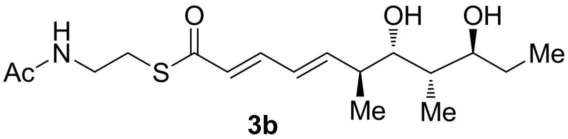

3b

Step 1: To a solution of the acid **31** 0.49 mmol, 0.23 g) in dry DMF (6.6 mL) at room temperature were added HOBt (0.64 mmol, 0.086 g) and *N*-(2-mercaptoethyl)acetamide (2.45 mmol, 0.26 mL). Then, the reaction was cooled to 0 ºC and a solution of DCC (0.64 mmol, 0.131 g) in dry DMF (2 mL) was added. The reaction was warmed to room temperature and stirred for 48 h. After this time, the reaction was quenched by the addition of saturated aqueous NH_4Cl solution (10 mL). The aqueous layer was extracted with EtOAc (3 x 20 mL). The combined organic layers were dried over $MgSO_4$, filtered and concentrated *in vacuum*. The crude mixture was purified by flash column chromatography (silica gel and hexanes/EtOAc, 70:30). *Rf* = 0.39 (30% EtOAc in hexanes). The presence of 1,3-dicyclohexylurea in the purified product **3b** was observed. Thus, the mixture of compounds was used in the following step. Step 2: To a stirring solution of the obtained mixture in THF (4.45 mL) in a polyethylene flask at 0 °C was added a solution of HF:pyridine (4.45 mL, 60-70% solution). The reaction was stirred for 3 days at room temperature and then portions of solid $NaHCO_3$ (4.0 g) were added. The aqueous layer was extracted with EtOAc (3 x 30 mL) and the combined organic layers were dried over $MgSO_4$, filtered and concentrated. The crude product was purified by flash chromatography in silica gel (15% $CHCl_3$/EtOAc) to give the diol **3b** (85 mg, 51%, for 2 steps) as colorless oil. R*f* 0.12 (70% EtOAc/hexanes). $[\alpha]^{20}_D$ –27.0 (c 1.07 MeOH). ^{1}H NMR (500 MHz, MeOD): 7.08 (dd, J_1 = 15.2 Hz, J_2 = 10.0 Hz, 1H), 6.15 (dd, J_1 = 15.2 Hz, J_2 = 7.3 Hz, 1H), 6.10 (dd, J_1 = 15.2 Hz, J_2 = 10.0 Hz, 1H), 6.01 (d, J = 15.2 Hz, 1H), 3.56 (dd, J_1 = 8.2 Hz, J_2 = 2.8 Hz, 1H), 3.17 (t, J = 6.8 Hz, 2H), 3.13 (m, 1H), 2.90 (t, J = 6.8

Hz, 2H), 2.30–2.23 (m, 1H), 1.74 (s, 3H), 1.51–1.40 (m, 2H), 1.29–1.20 (m, 1H), 0.83–0.78 (m, 6H), 0.73 (d, J = 7.0 Hz, 3H) ppm. ^{13}C NMR (125 MHz, MeOD): 191.0, 173.4, 151.3, 143.0, 129.3, 127.3, 76.2, 75.5, 42.5, 40.8, 40.2, 28.9, 28.1, 22.4, 17.1, 10.5, 10.1 ppm. ^{13}C NMR DEPT 135 (62.5 MHz, $CDCl_3$): 129.0, 113.5, 76.6, 72.7, 72.2, 69.1, 55.1, 37.8, 33.6, 27.6, 24.8, 23.5, 13.9, 11.7, 10.4 ppm. IR υ_{max} (film): 3286, 2965, 2932, 2676, 1653, 1629, 1541, 1457, 1373, 1288, 1021, 995, 967, 814 cm^{-1}.

HRMS calc. For $C_{17}H_{29}NO_4SH^+$: 344.1890. Found: 344.1907. Error (ppm): 4.94.

(2*E*,4*E*,6*S*,7*S*,8*S*,9*S*)-7,9-dihydroxy-6,8-dimethylundeca-2,4-dienoic acid (3a)

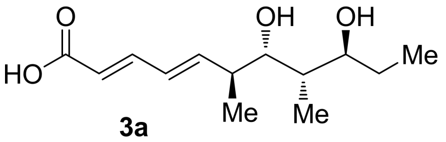

3a

^{1}H NMR (250 MHz, MeOD): 7.03 (dd, J_1 = 15.6 Hz, J_2 = 10.6 Hz, 1H), 6.22 (dd, J_1 = 15.6 Hz, J_2 = 10.6 Hz, 1H), 6.02 (dd, J_1 = 15.6 Hz, J_2 = 8.1 Hz, 1H), 5.81 (d, J = 15.6 Hz, 1H), 3.69 (dd, J_1 = 8.1 Hz, J_2 = 2.8 Hz, 1H), 3.52–3.44 (m, 1H), 2.45–2.29 (m, 1H), 1.74–1.35 (m, 2H), 1.48–1.36 (m, 1H), 1.0–0.89 (m, 9H) ppm. ^{13}C NMR (125 MHz, $CDCl_3$): 176.2, 145.1, 142.4, 130.3, 127.3, 76.3, 75.6, 42.2, 40.8, 28.1, 17.4, 10.5, 10.1 ppm. IR υ_{max} (film): 3380, 2967, 2979, 1687, 1640, 1460, 1412, 1382, 1303, 1263, 1150, 1001, 968 cm^{-1}.

HRMS calc. For $C_{13}H_{22}O_4Na^+$: 265.1410. Found: 265.1403. Error (ppm): –2.83.

4. NMR and HRMS Elucidation of the Compounds Generated by Ela-TE.

General Remarks: NMR spectra were recorded on a Bruker 500 MHz DCH Cryoprobe Spectrometer except for # which was recorded on a Bruker 500 MHz TCI Cryoprobe Spectrometer. Chemical shifts are expressed in parts per million (ppm) on the δ scale, referenced to C**H**Cl_3 at δ_H 7.26 (^{1}H) and **C**HCl_3 at δ_C 77.0 (^{13}C), or CD_3OD, referenced to C**H**$_3$OD at δ_H 3.3 (^{1}H) and **C**H_3OD at δ_C 49.3 (^{13}C). Where ^{1}H signals were obscured by other signals or contaminants they were obtained from HSQC experiments (indicated by 'obs').

4.1. Compound 4 $^{\#}$.

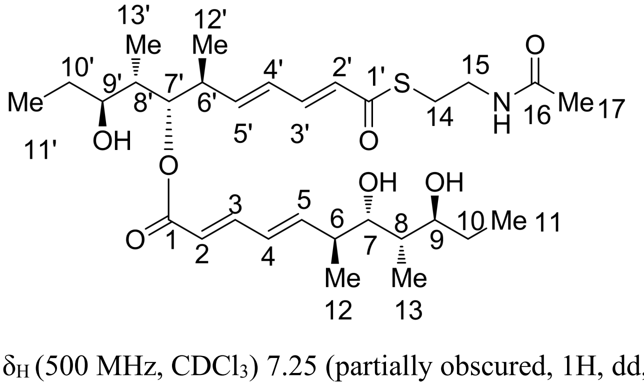

δ_H (500 MHz, $CDCl_3$) 7.25 (partially obscured, 1H, dd, 15.0, 11.2 Hz, H3), 7.13 (2H, dd, 15.1, 10.6 Hz, H3'), 6.26 (1H, dd, 15.5, 10.8 Hz, H4), 6.17-6.02 (3H, m, H4', H5, H5'), 6.07 (1H, d, 15.2 Hz, H2'), 5.77 (1H, d,

15.5 Hz, H2), 5.14 (1H, dd, 9.4, 1.8 Hz, H7’), 3.76 (1H, dd, 9.5, 2.0 Hz, H7), 3.57 (1H, m, H9), 3.45 (2H, m, H15), 3.10 (2H, m, H14), 3.04 (1H, m, H9’), 2.64 (1H, m, H6’), 2.41 (1H, m, H6), 1.95 (3H, s, H17), 1.74 (obs, H8), 1.71 (obs, H8’), 1.67 (obs, H10), 1.59 (obs, H10’), 1.35 (obs, H10), 1.05 (3H, d, 6.9 Hz, H12’), 1.01 (3H, d, 7.1 Hz, H12), 0.99-0.95 (9H, m, H11, H11’, H13’), 1.86 (3H, d, 7.0 Hz, H13); δ_C (125 MHz, CDCl$_3$, observed indirectly by HSQC/HMBC experiments) 190.4 (C, C1’), 170.3 (C, C16), 167.9 (C, C1), 148.2 (CH, C5 or C5’), 148.0 (CH, C5 or C5’), 146.0 (CH, C3), 141.5 (CH, C3’), 128.8 (CH, C4 and C4’), 126.8 (CH, C2’), 119.1 (CH, C2), 77.6 (CH, C9), 76.3 (CH, C7’), 74.0 (CH, C7), 72.9 (CH, C9’), 41.2 (CH, C6), 40.1 (CH, C8), 39.8 (CH, C6’), 39.8 (CH$_2$, C15), 37.4 (CH, C8’), 28.2 (CH$_2$, C14), 28.1 (CH$_2$, C10’), 26.7 (CH$_2$, C10), 23.2 (CH$_3$, C17), 16.4 (CH$_3$, C12’), 16.2 (CH$_3$, C12), 10.2 (CH$_3$, C11, C11’ or C13’), 10.2 (CH$_3$, C11, C11’ or C13’), 10.1 (CH$_3$, C11, C11’ or C13’), 9.5 (CH$_3$, C13);

HRMS calc. for $C_{30}H_{49}N_1O_7S_1Na^+$: 590.3122. Found: 590.3104. Error (ppm): –2.99.

See the NMR spectra in Supporting Information 5.1.

4.2. Compound 5.

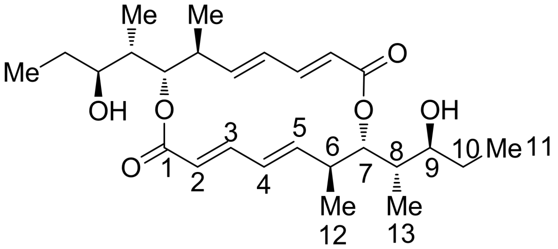

δ_H (500 MHz, CDCl$_3$) 6.97 (2H, ddd, 15.5, 11.2, 0.8 Hz, H3), 6.10 (2H, dd, 15.2, 11.2 Hz, H4), 5.66 (2H, d, 15.5 Hz, H2), 5.65 (2H, dd, 15.2, 9.6 Hz, H5), 4.95 (2H, dd, 10.3, 2.0 Hz, H7), 3.18 (2H, td, 8.8, 2.7 Hz, H9), 2.51 (2H, m, H6), 1.78 (2H, m, H8), 1.70 (2H, m, H10), 1.37 (2H, m, H10), 1.02 (6H, d, 6.8 Hz, H12), 0.98 (6H, t, 7.5 Hz, H11), 0.89 (6H, d, 7.0 Hz, H13); δ_C (125 MHz, CDCl$_3$) 169.3 (C, C1), 145.0 (CH, C3), 144.4 (CH, C5), 131.7 (CH, C4), 121.2 (CH, C2), 77.8 (CH, C7), 73.3 (CH, C9), 41.1 (CH, C6), 38.8 (CH, C8), 29.7 (CH$_2$, C10), 15.0 (CH$_3$, C12), 10.0 (CH$_3$, C11), 9.4 (CH$_3$, C13);

HRMS calc. for $C_{26}H_{40}O_6H^+$: 449.2898. Found: 449.2886. Error (ppm): –2.64.

See the NMR spectra in Supporting Information 5.2.

4.3. Compound 6.

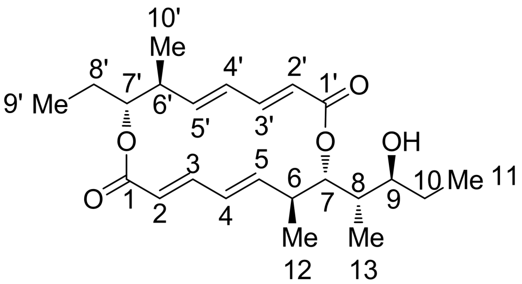

δ_H (500 MHz, CD_3OD) 6.884 (1H, ddd, 15.4, 11.1, 0.6 Hz, H3 or H3'), 6.881 (1H, ddd, 15.4, 11.1, 0.7 Hz, H3 or H3'), 6.13 (1H, dd, 14.9, 11.1 Hz, H4 or H4'), 6.12 (1H, dd, 14.9, 11.1 Hz, H4 or H4'), 5.70 (1H, d, 15.4 Hz, H2 or H2'), 5.69 (1H, d, 15.4 Hz, H2 or H2'), 5.66 (1H, dd, 15.0, 10.0 Hz, H5 or H5'), 5.62 (1H, dd, 15.0, 10.0 Hz, H5 or H5'), 5.16 (1H, dd, 10.4, 1.5 Hz, H7), 4.63 (1H, td, 9.7, 2.7 Hz, H7'), 3.20 (1H, td, 8.7, 2.9 Hz, H9), 2.54 (1H, m, H6), 2.31 (1H, m, H6'), 1.87 (1H, m, H8'), 1.82 (1H, m, H8), 1.65 (1H, dqd, 13.9, 7.4, 2.9 Hz, H10), 1.53 (1H, m, H8'), 1.33 (1H, m, H10), 1.06 (3H, d, 6.6 Hz, H10'), 1.04 (3H, d, 6.7 Hz, H12), 0.951 (3H, t, 7.3 Hz, H11 or H9'), 0.949 (3H, t, 7.3 Hz, H11 or H9'), 0.94 (3H, d, 7.0 Hz, H13); δ_C (125 MHz, CD_3OD) 169.9 (C, C1/C1'), 146.7 (CH, C3/C3'), 146.0 (CH, C5 or C5'), 145.8 (CH, C5 or C5'), 132.5 (CH, C4/C4'), 122.7 (CH, C2/C2'), 80.1 (CH, C7'), 78.3 (CH, C7), 74.8 (CH, C9), 46.0 (CH, C6'), 42.9 (CH, C6), 40.1 (CH, C8), 28.2 (CH_2, C10), 25.7 (CH_2, C8'), 16.2 (CH_3, C12 or C10'), 16.0 (CH_3, C12 or C10'), 10.3 (CH_3, C13, C11 or C9'), 10.2 (CH_3, C13, C11 or C9'), 9.8 (CH_3, C13, C11 or C9');

HRMS calc. for $C_{23}H_{34}O_5Na^+$: 413.2298. Found: 413.2284. Error (ppm): −3.45.

See the NMR spectra in Supporting Information 5.3.

5. Selected NMR Spectra.

5.1. Compound 4.

^{1}H NMR

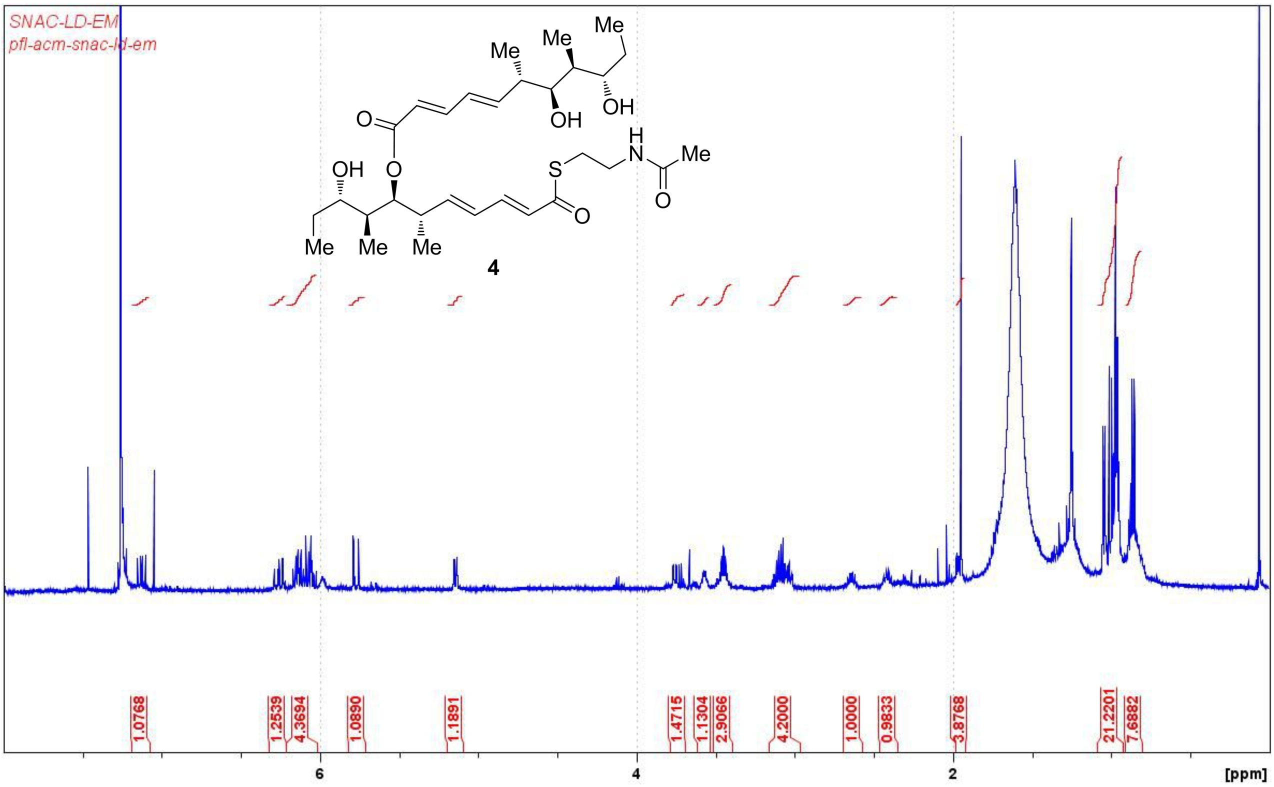

COSY NMR

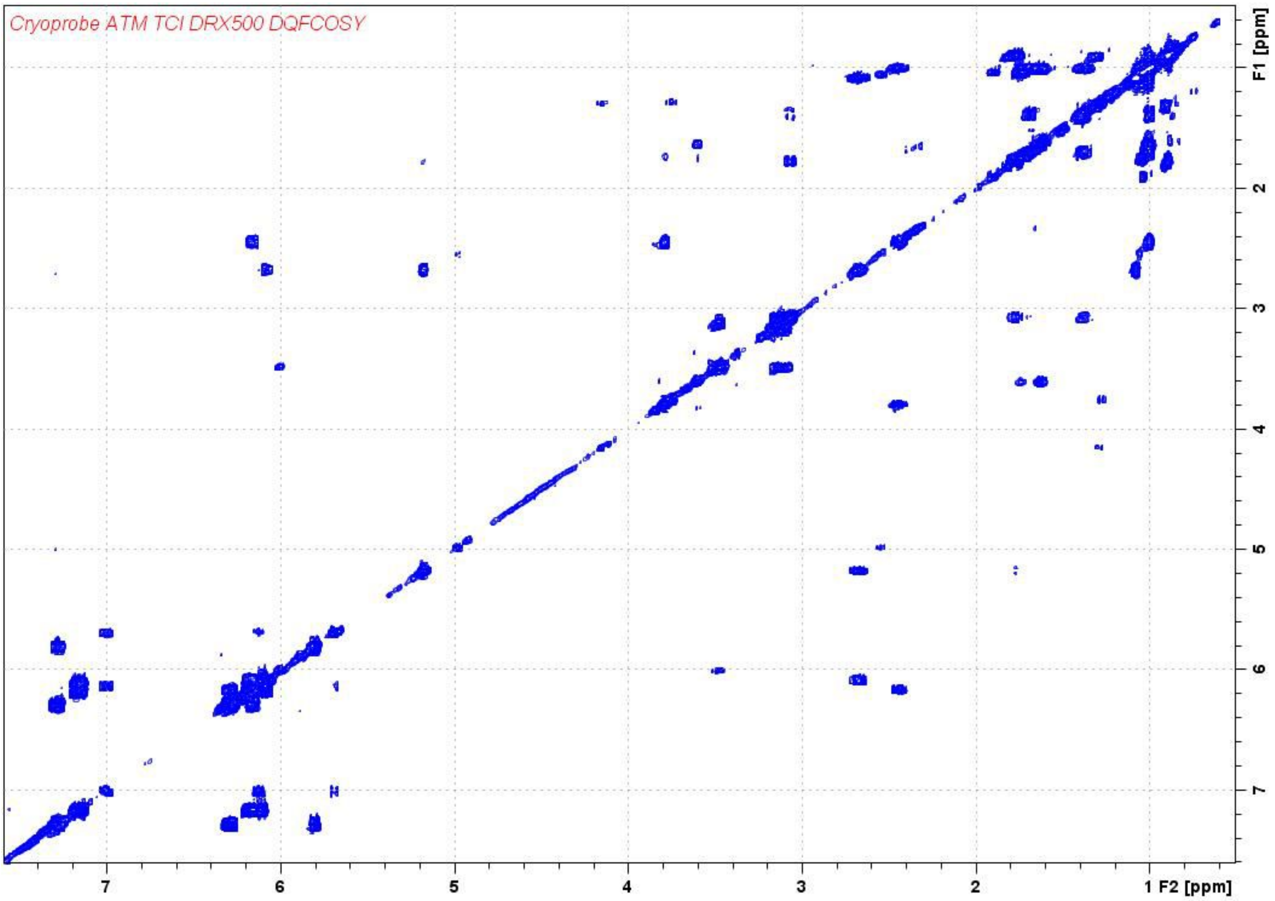

HSQC NMR

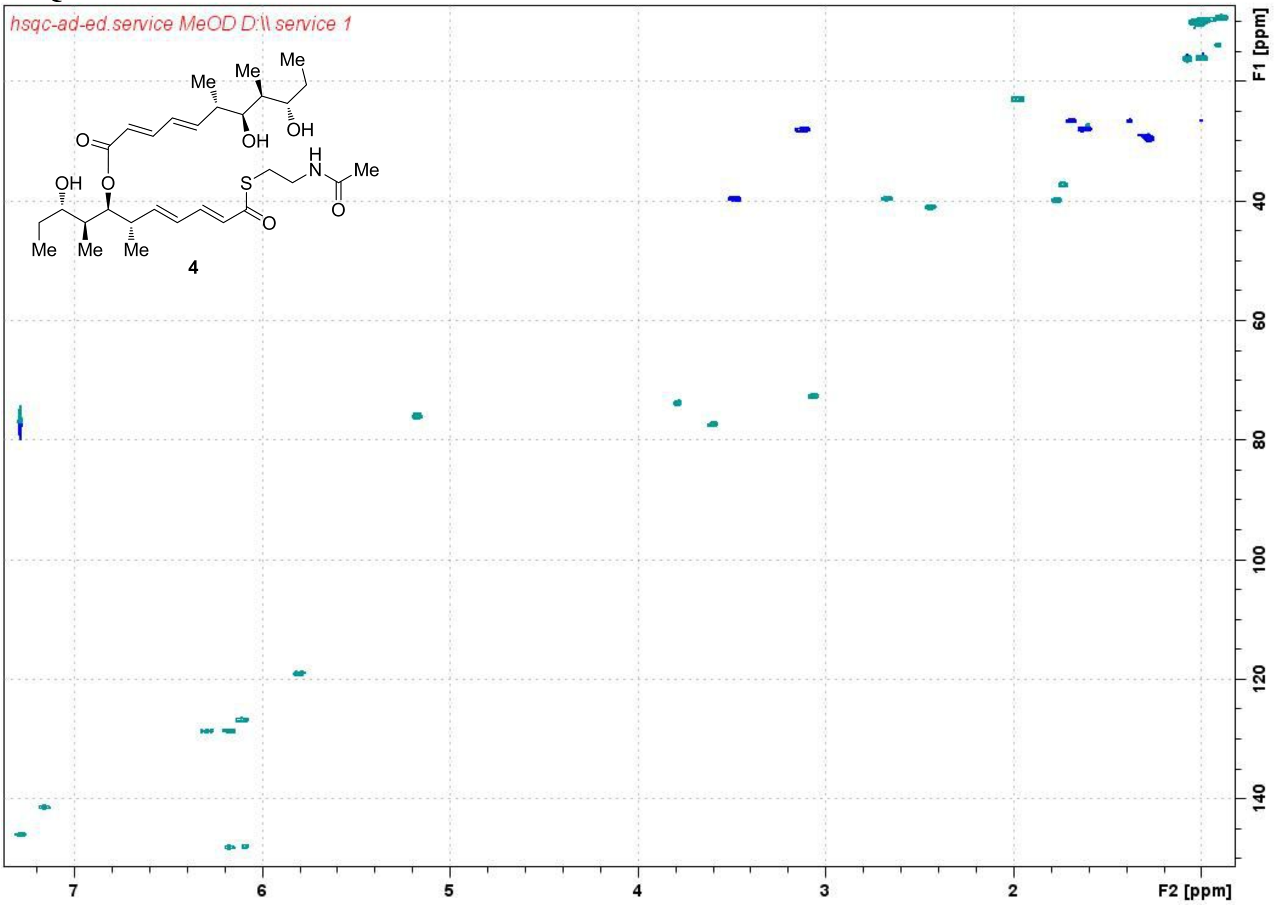

HMBC NMR

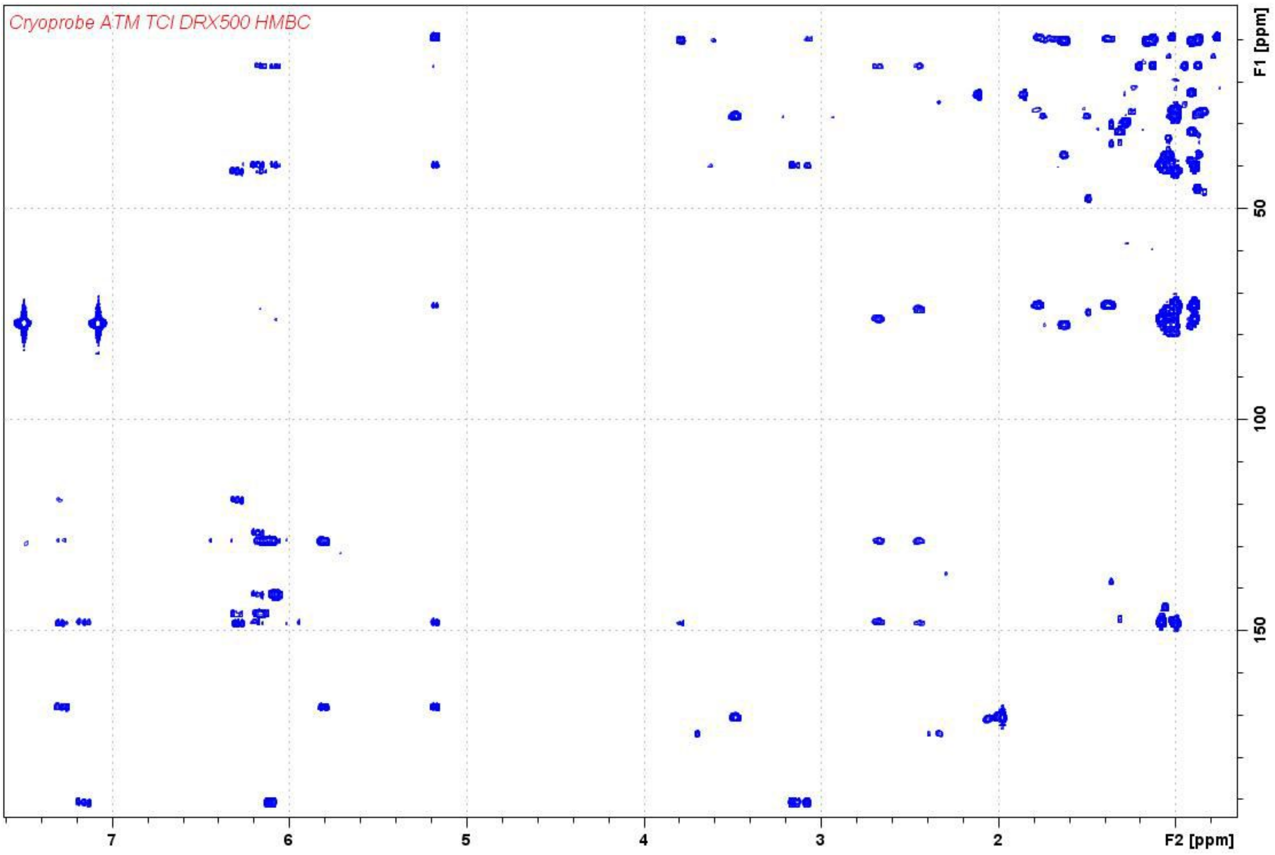

5.2. Compound 5.

^{1}H NMR

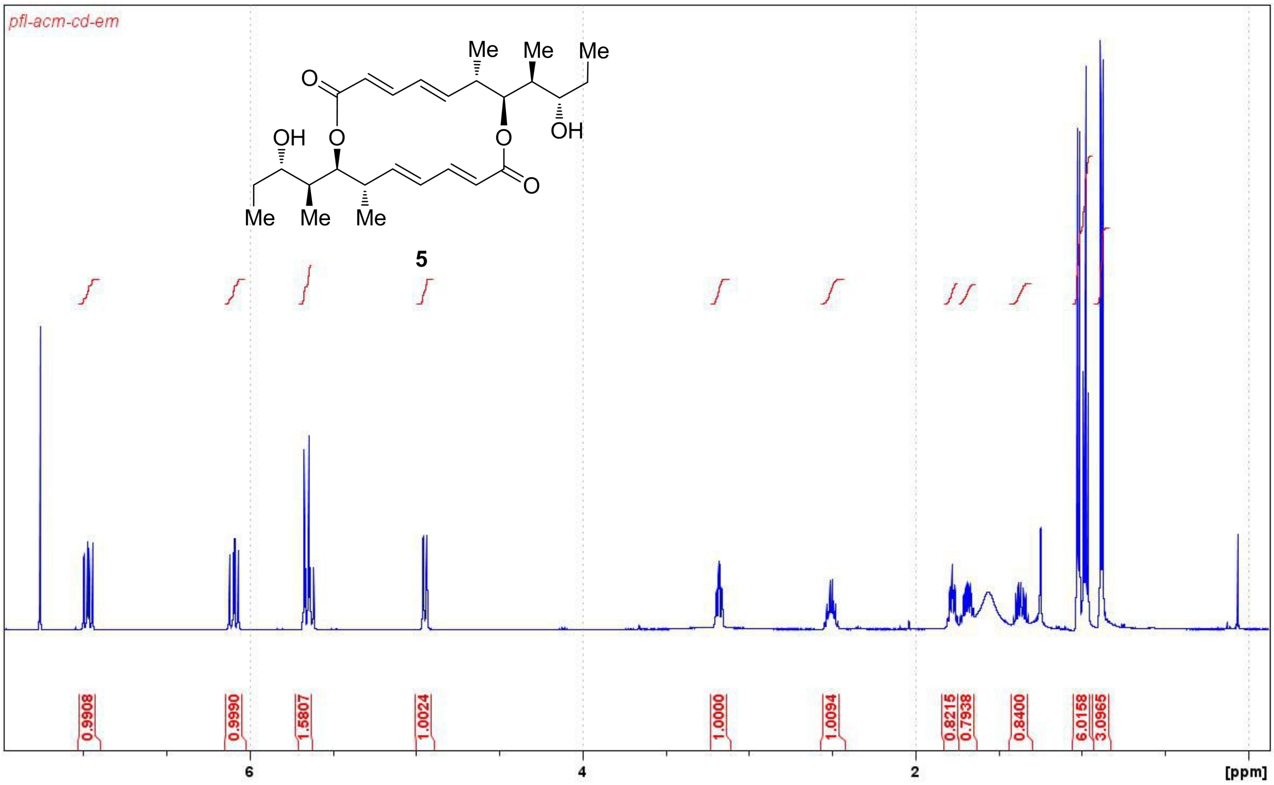

^{13}C NMR

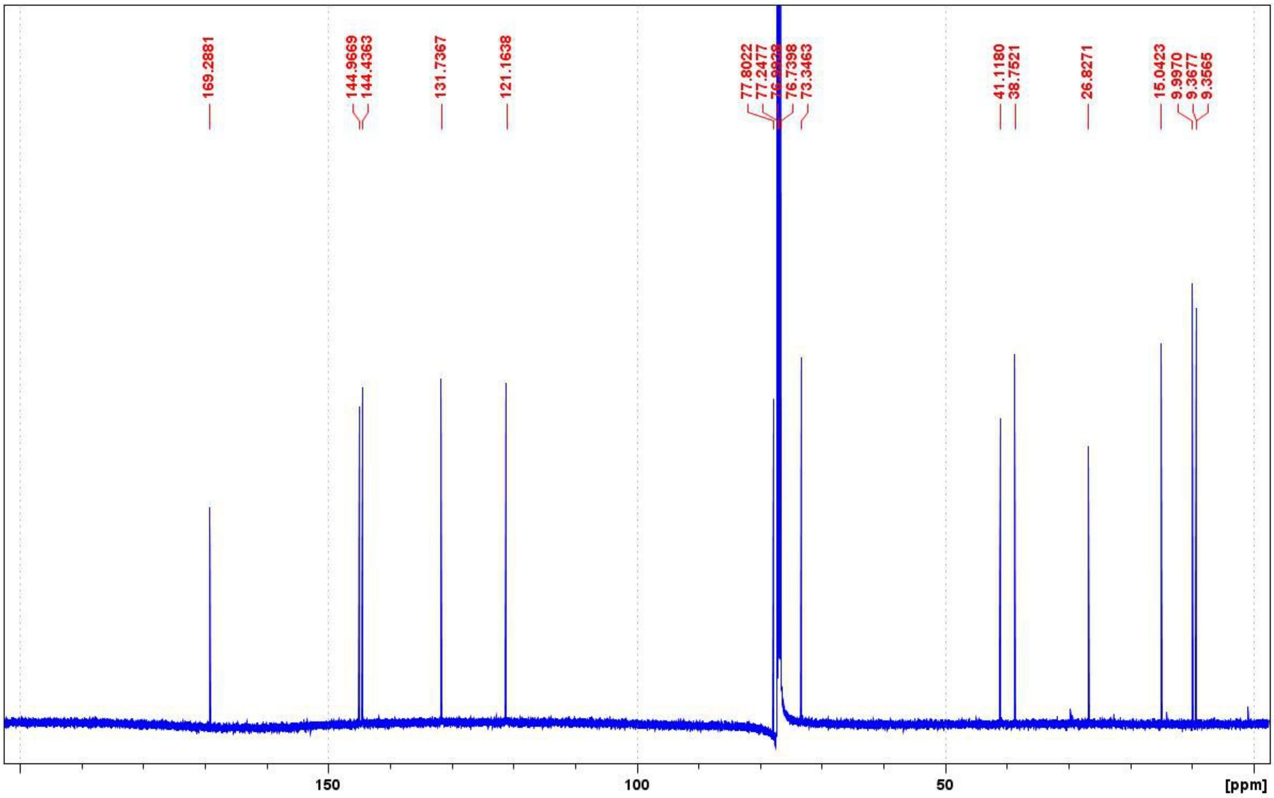

COSY NMR

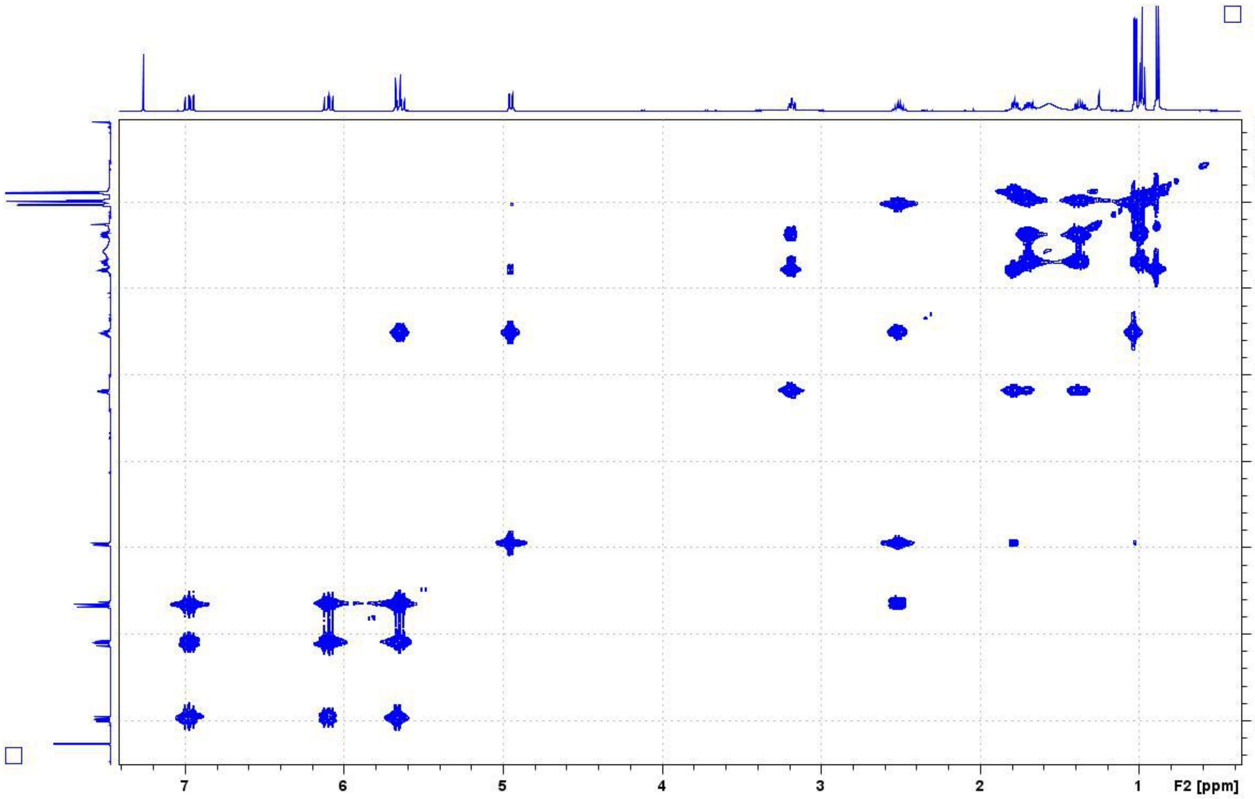

HSQC NMR

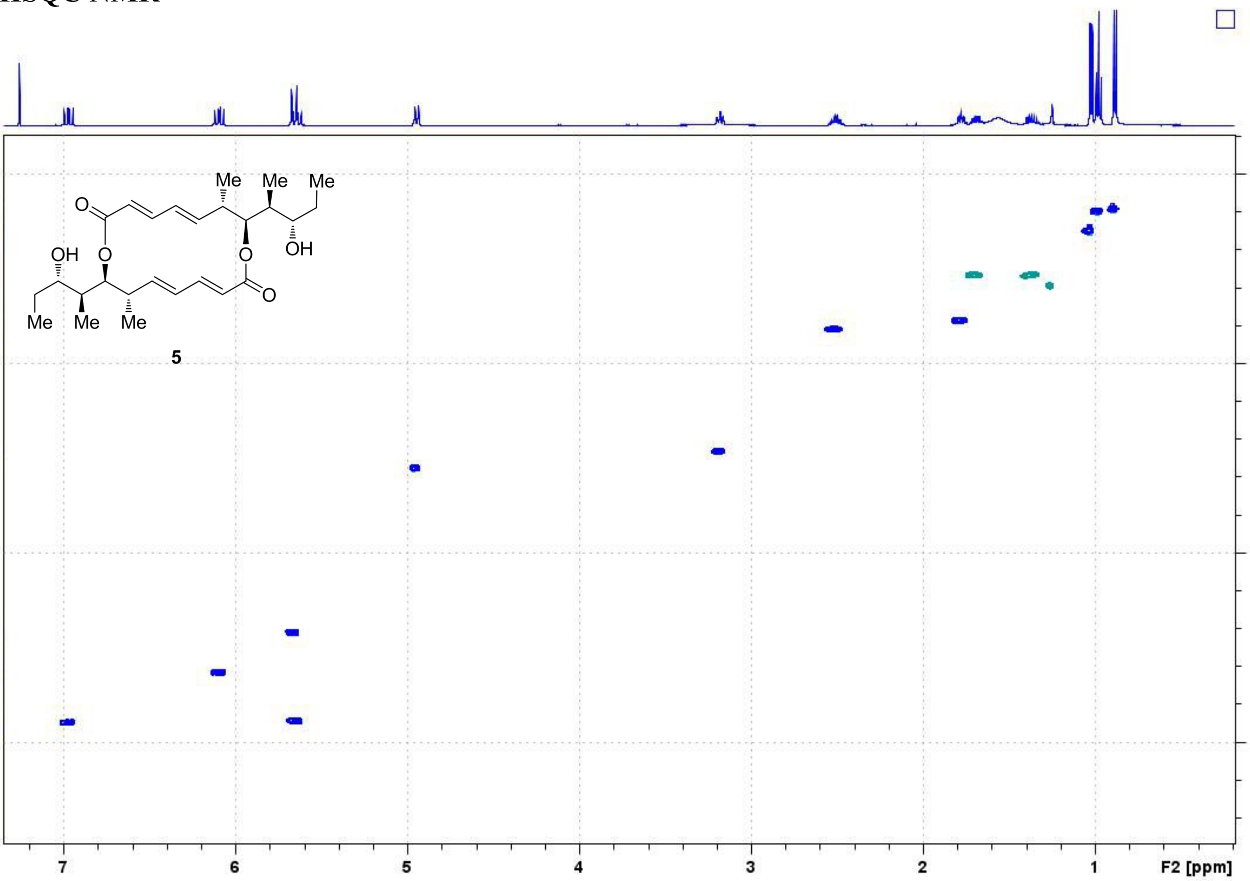

HMBC NMR

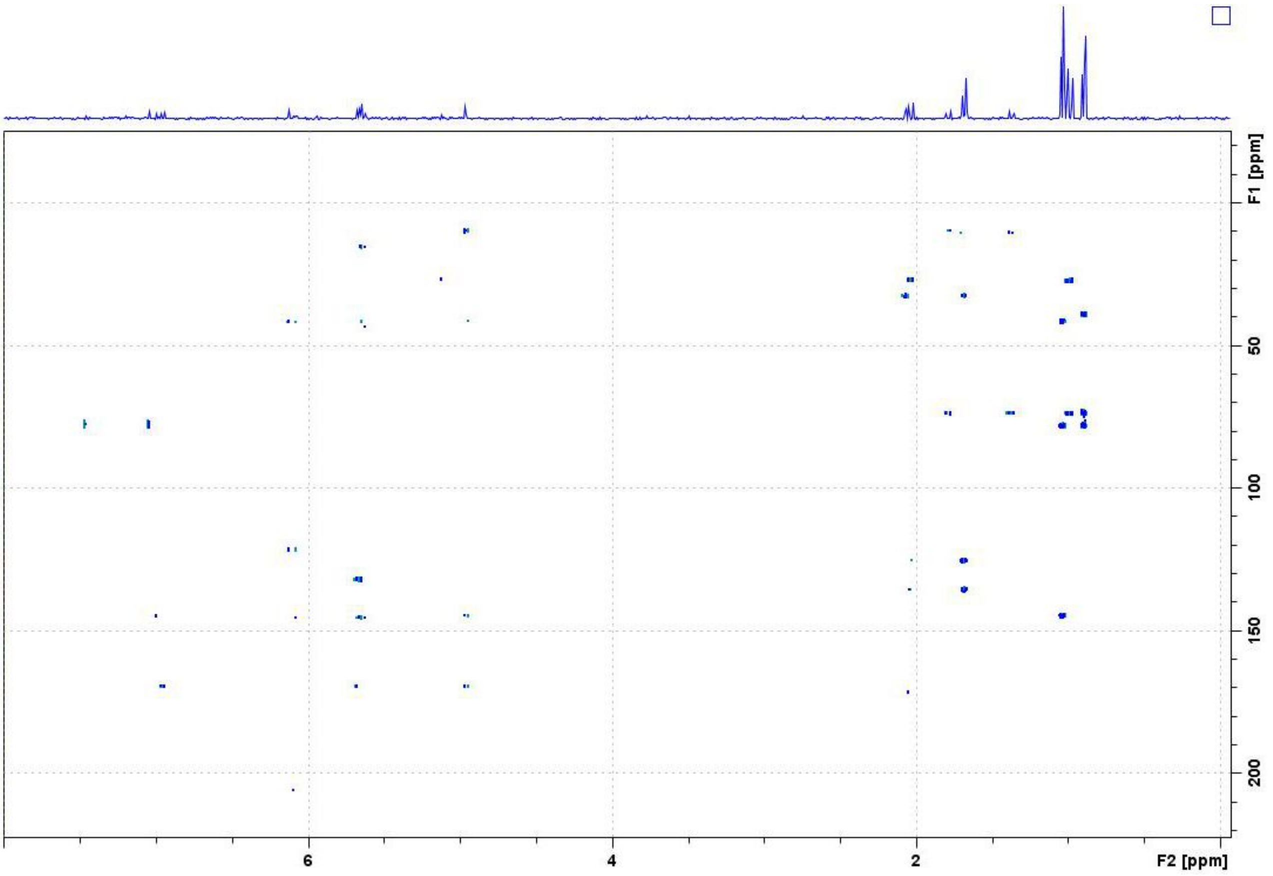

5.3. Compound 6.

^{1}H NMR

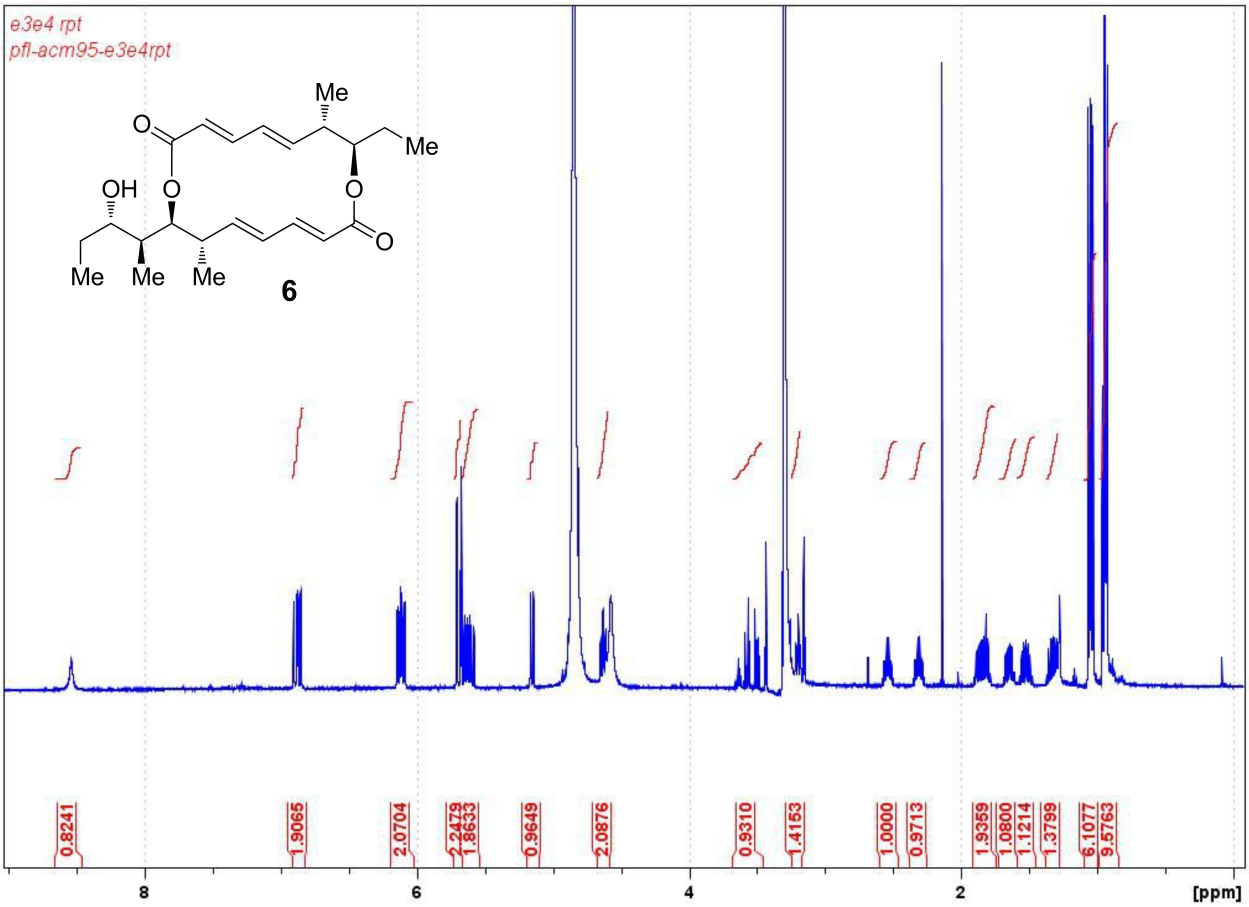

^{13}C NMR

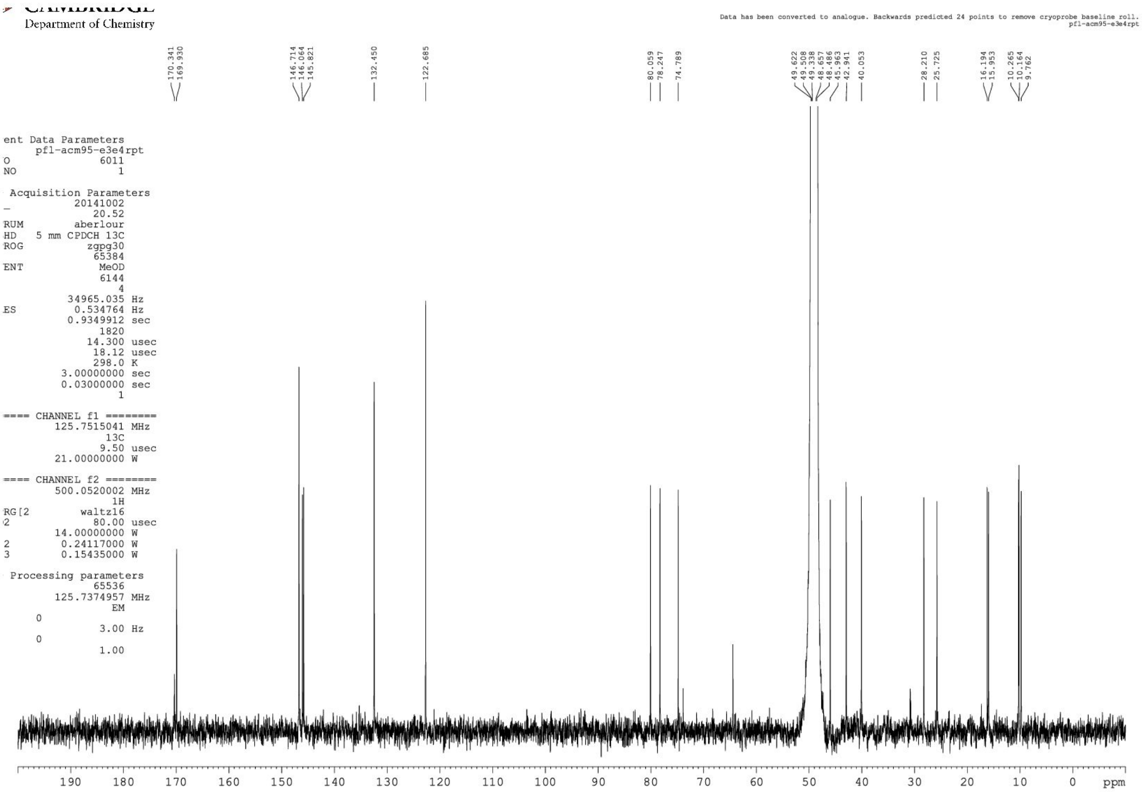

COSY NMR*

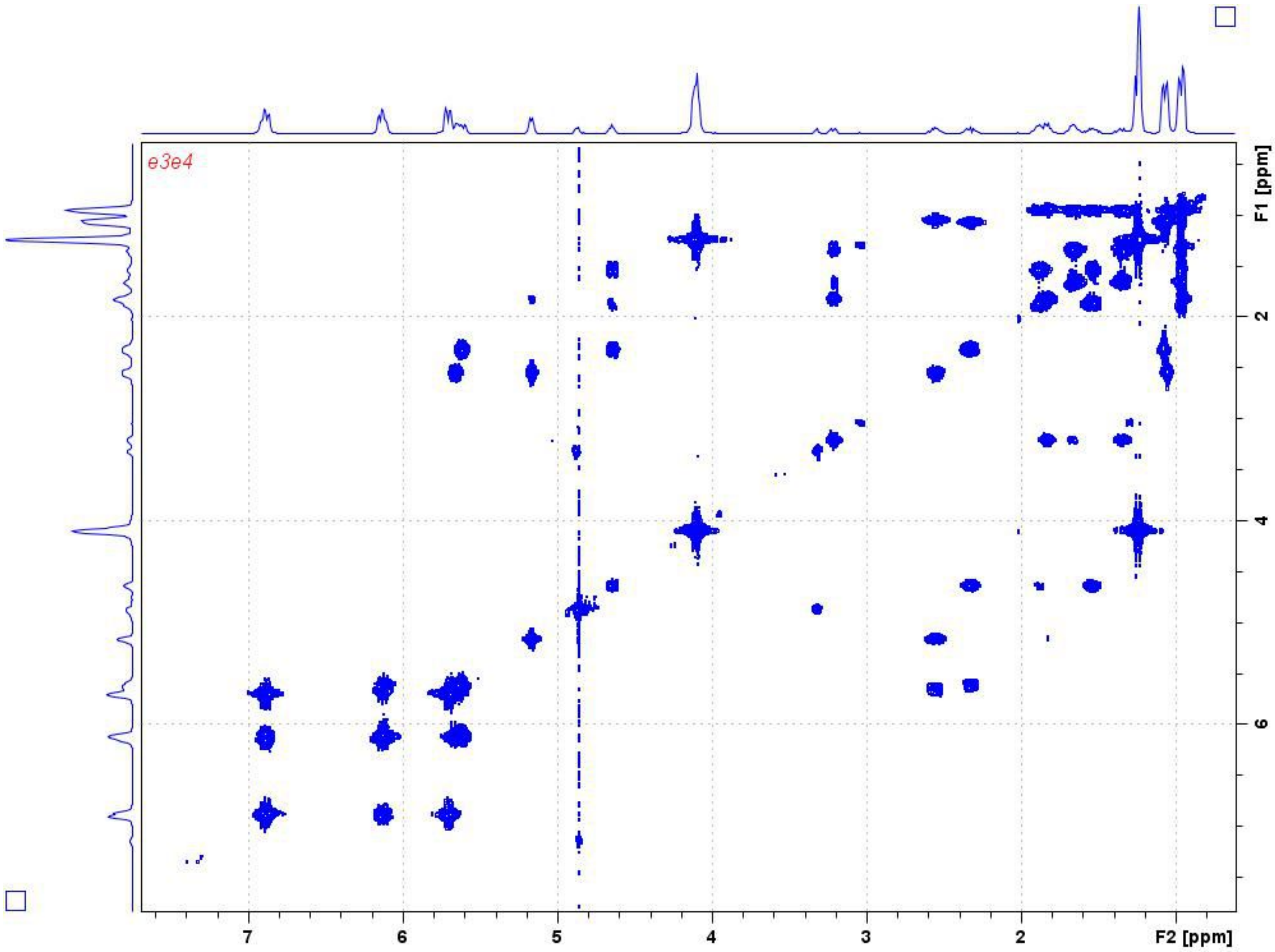

HSQC NMR*

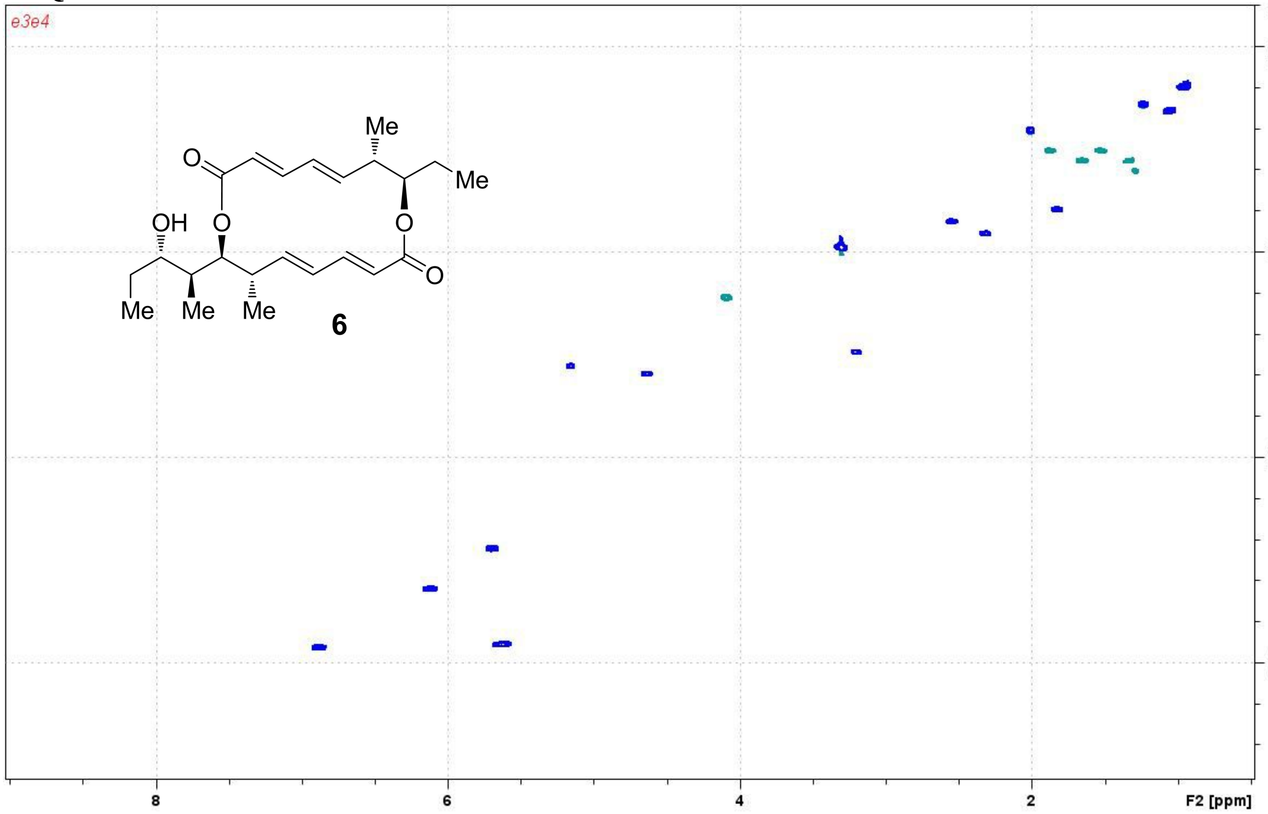

HMBC NMR*

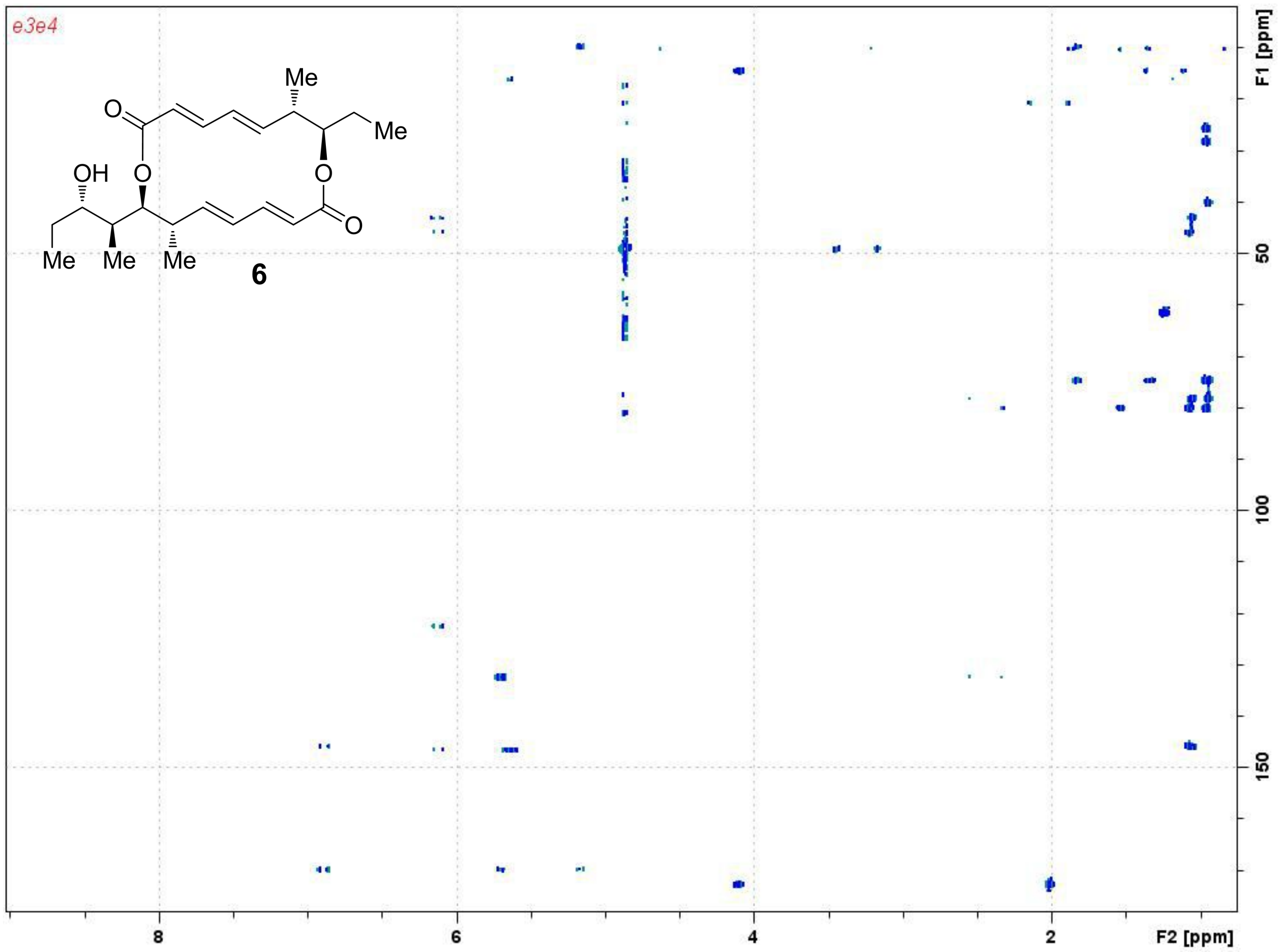

* 2D NMR data contain signals arising from ethyl acetate contamination

5.4. Compound 3b.

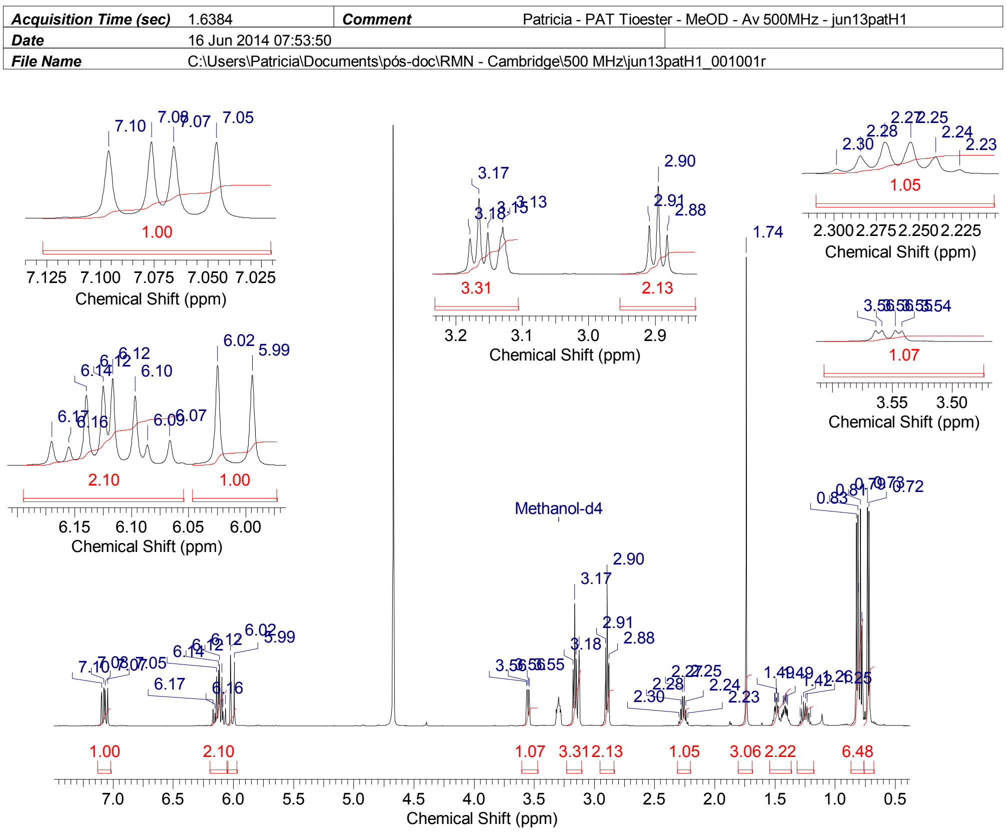

Spectrum of ^{1}H NMR of the compound **3b** in MeOD, 500 MHz, 25 °C.

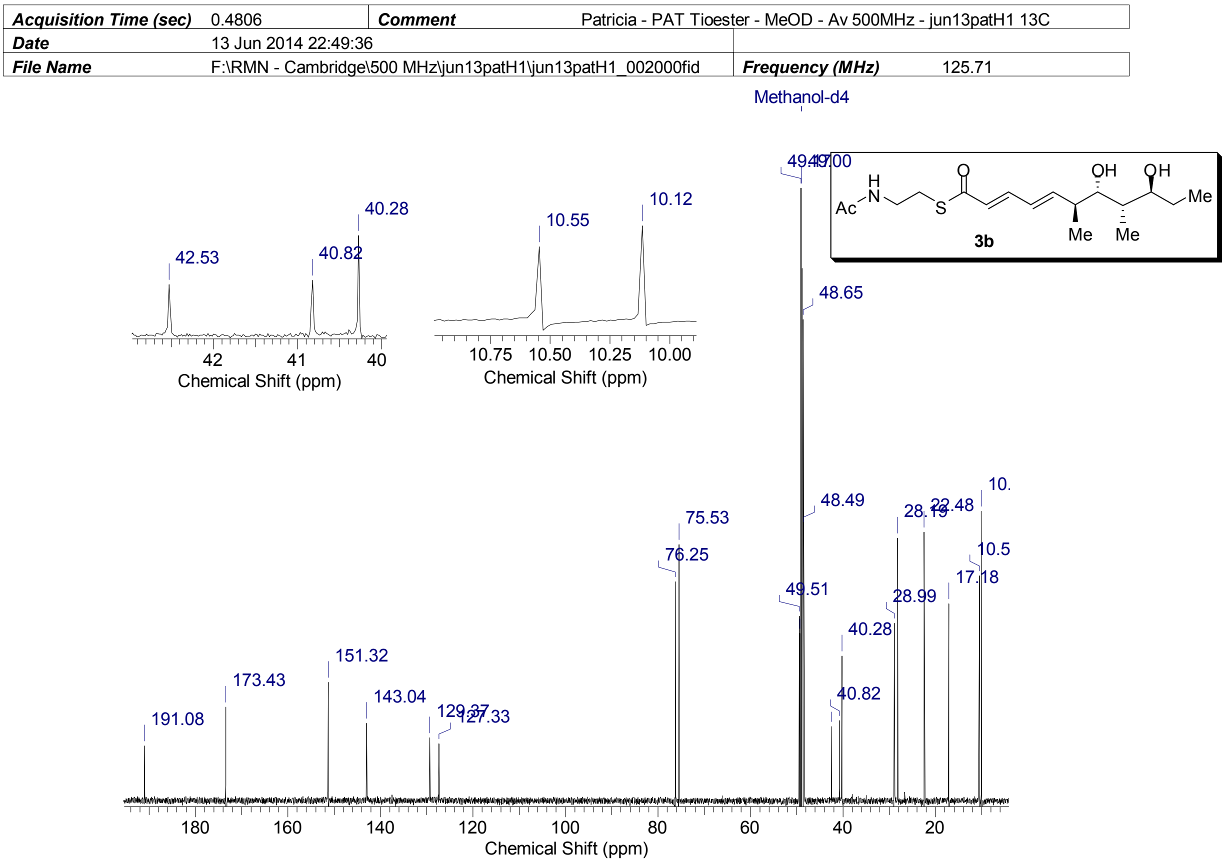

Spectrum of ^{13}C NMR of the compound **3b** in MeOD, 125 MHz, 25 °C.

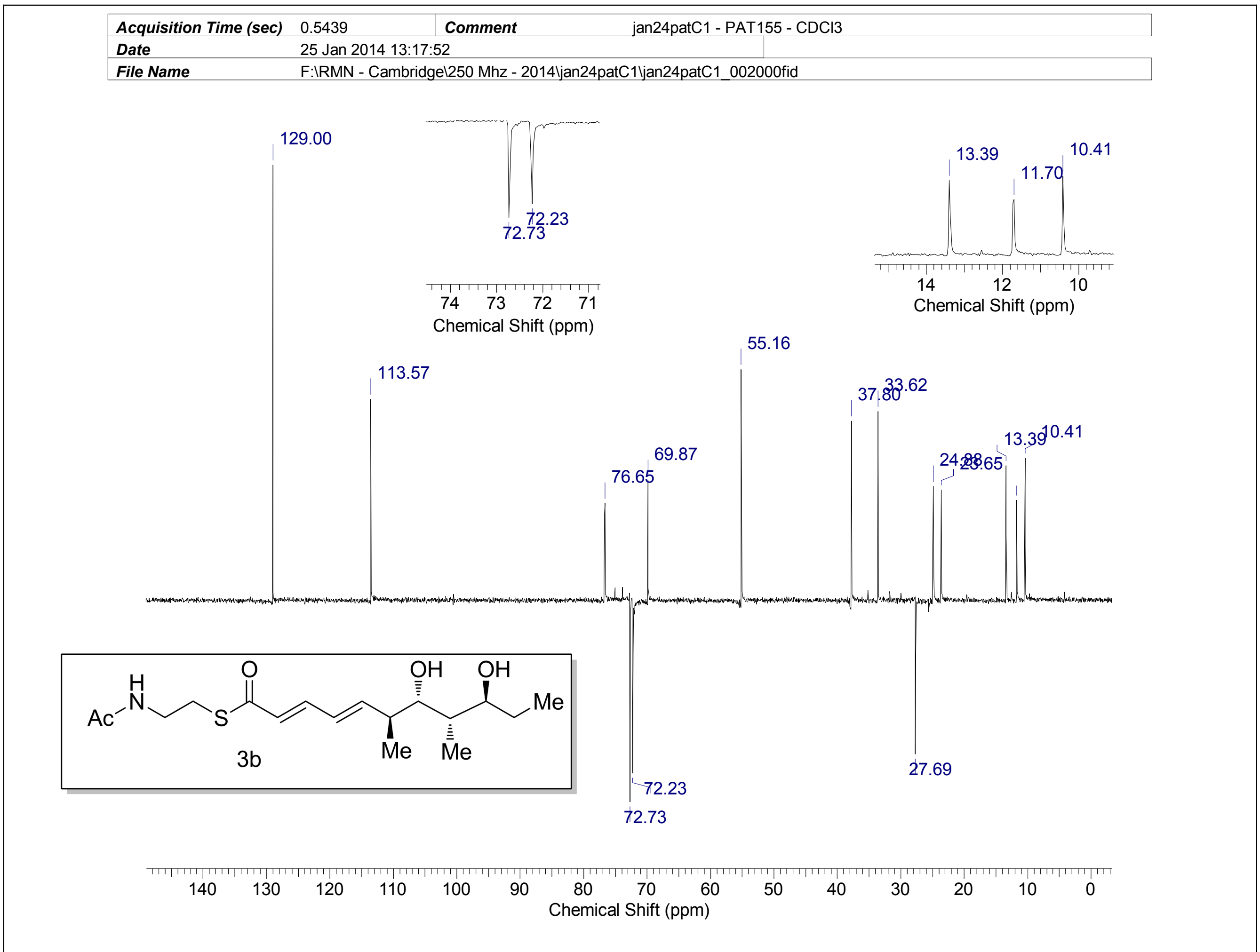

Spectrum of ^{13}C NMR (DEPT 135) of the compound **3b** in $CDCl_3$, 62.5 MHz, 25 °C.

5.5. Compound 2b.

Acquisition Time (sec)	2.6564	Date	19 Aug 2013 07:59:44		
File Name	C:\Users\Patricia\Documents\pós-doc\RMN - Cambridge\600 MHz\ago16patC1_002001r				
Frequency (MHz)	600.17	Nucleus	1H	Number of Transients	32

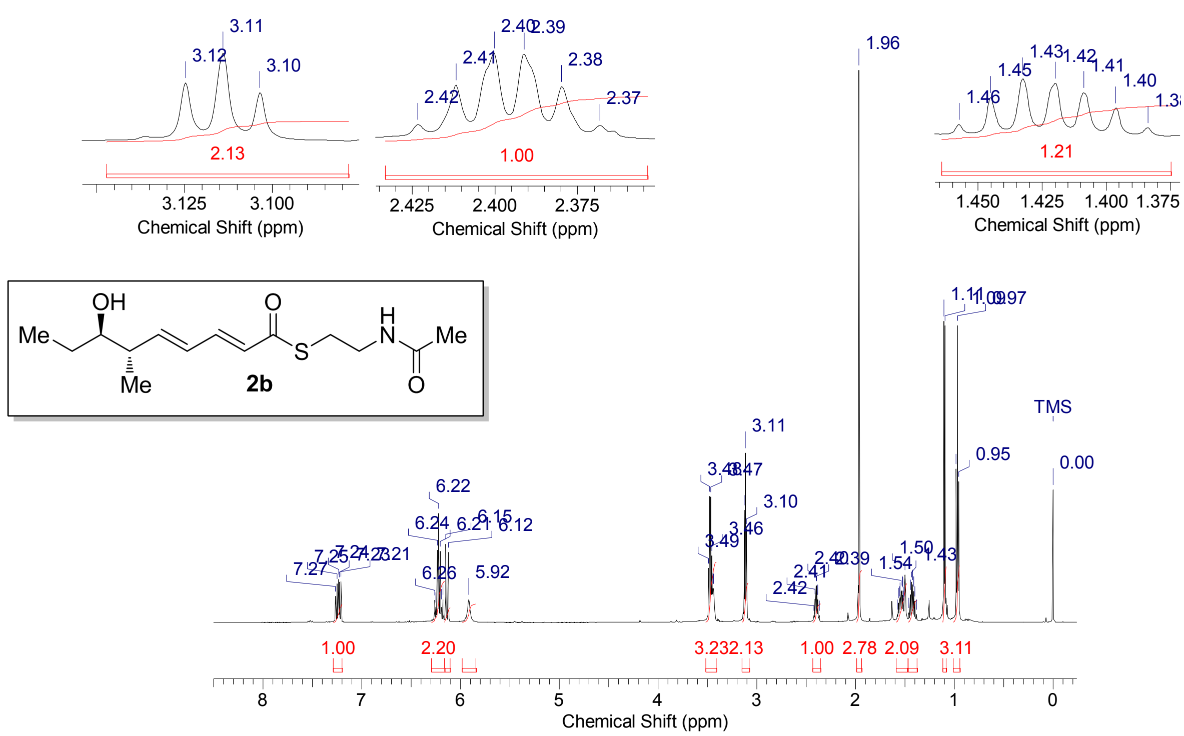

Spectrum of ^{1}H NMR of the compound **2b** in $CDCl_3$, 600 MHz, 25 ºC.

Acquisition Time (sec)	0.4544	Date	19 Aug 2013 07:53:20		
File Name	C:\Users\Patricia\Documents\pós-doc\RMN - Cambridge\600 MHz\ago16patC1_001001r				
Frequency (MHz)	150.91	Nucleus	13C	Number of Transients	20480

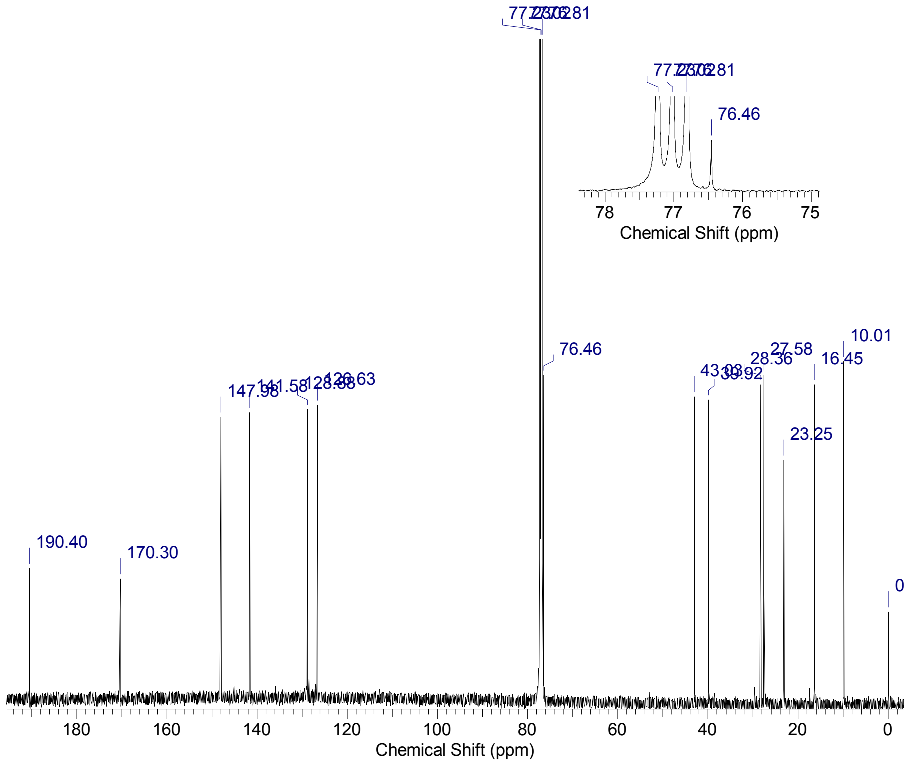

Spectrum of ^{13}C NMR of the compound **2b** in $CDCl_3$, 150 MHz, 25 ºC.

6. Supplementary References

[1] S. Grabley, P. Hammann, W. Raether, J. Wink, A. Zeeck, *J Antibiot*. **1990**, *43*, 639-647.

[3] L. Tran, M. Tosin, J. B. Spencer, P.F. Leadlay, K.J. Weissman, *Chembiochem*. **2008**, *9*, 905-915.

[4] J. W. Giraldes, D. L. Akey, J. D. Kittendorf, D. H. Sherman, J. L. Smith, R.A. Fecik, *Nat Chem Biol*. **2006**, *2*, 531-536.

[5] J. B. Scaglione , D. L. Akey, R. Sullivan, J. D. Kittendorf, C. M. Rath, E. S. Kim, J. L. Smith, D. H. Sherman. *Angew Chem Int Ed Engl*. **2010**, *49*, 5726-5730.

[6] S. C. Tsai, L. J. Miercke, J. Krucinski, R. Gokhale, J. C. Chen, P. G Foster, D. E. Cane, C. Khosla, R. M. Stroud. *Proc Natl Acad Sci*. **2001**, *98*, 14808-14813.

[7] T. M. Laue, B. D. Shah, T. M. Ridgeway, S. L. Pelletier, Royal Society of Chemistry Ed: S. E. Harding, A. J. Rowe, J. C. Horton, **1992**, pp. 90-125.

[8] P. Schuck, *Biophys J,* **2000**, *78*, 1606-1619.

[9] D. G. Gibson, L. Young, R. Y. Chuang, J. C. Venter, C. A. Hutchison 3rd, H. O. Smith, *Nat Methods*. **2009**, 6, 343-345.

[10] G. Ashley, I. C.-W. Chan-Kai, M. A. Burlingame, patent WO 2000044717 A2 20000803, **2000**.

[11] L. C. Dias, C. C. Perez, *Eur. J. Org. Chem.* **2013**, 2930-2939.

[12] a) S. D. Rychnovsky, B. Rogers, G. Yang, *J. Org. Chem.* **1993**, *58*, 3511–3516; b) S. D. Rychnovsky, D. J. Skalitzky, Tetrahedron Lett. 1990, 31, 945–948; c) S. D. Rychnovsky, B. N. Rogers, T. I. Richardson, *Acc. Chem. Res.* **1998**, *31*, 9–17; d) D. A. Evans, D. L. Rieger, J. R. Gage, *Tetrahedron Lett.* **1990**, *31*, 7099–7100. For a theoretical study, see: e) C. F. Tormena, L. C. Dias, R. Rittner, *J. Phys. Chem. A* **2005**, *109*, 6077–6082.
